# Microbeam SAXS Reveals Flow-Induced Nanostructural Heterogeneity and Tilted Domains in 3D-Printed Block Copolymers

**DOI:** 10.1021/acs.macromol.6c01113

**Published:** 2026-08-13

**Authors:** Alice S. Fergerson, Emily C. Davidson

**Affiliations:** Department of Chemical and Biological Engineering, Princeton University, Princeton, New Jersey 08544, United States

## Abstract

The nozzle geometries associated with extrusion-based 3D printing impart significant spatial heterogeneities in both the type (shear vs extension) and rate of the flows applied during extrusion. For polymeric and nanostructured inks, these heterogeneous flow histories influence the underlying structure at the nano- to microscale. We previously demonstrated that HOT-DIW 3D printing can orient the nanostructure of a cylinder-forming block copolymer along a programmed print path, enabling tunable mechanical anisotropy. To probe the subfilament heterogeneities in nanostructure that arise in these oriented materials from the spatially varied flow histories, we apply scanning microbeam small-angle X-ray scattering (SM-SAXS). From this data, we identify a flow-rate-dependent emergence of off-axis tilted domains, which develop upon thermal annealing. We propose that application of shear followed by extension during 3D printing imparts asymmetric stretching of bridging chain conformations. Relaxation of this trapped stress upon thermal annealing imparts a torque on the shortened polystyrene domains which drives the formation of long-range ordered cylinders at an angle offset to the print direction. We show that the distribution of tilts within 3D-printed filaments is consistent with the variability in printed filament stiffness. Ultimately, these flow-structure insights highlight the need to understand how “real” mixed flows of complex fluids such as block copolymer melts drive the assembly, structure, and properties of printed materials.

## Graphical abstract

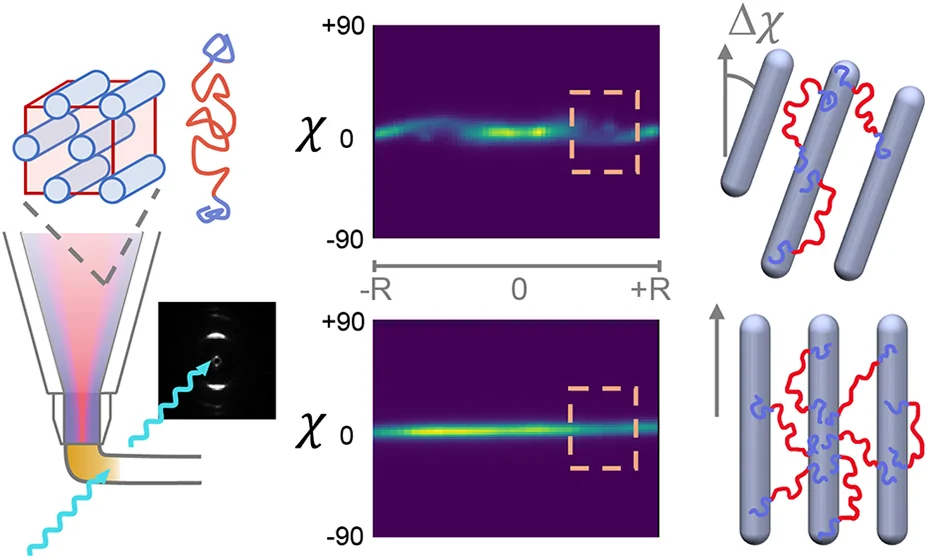

## Introduction

3D printing is rapidly emerging as a uniquely customizable manufacturing technique with the ability to control material structure and function from the most local length scales (1–100 nm) to truly macroscopic scales (cm-m). This ability to fabricate hierarchically ordered (e.g., multiscale architected^1–3^ ) structures transcends the capabilities of traditional manufacturing techniques; through 3D printing, one can adjust processing conditions “on-the-fly” to locally tune the material structure and properties to approach the multiscale structural complexity observed throughout biological systems.^4–7^ Materials which exhibit nanostructures with tunable anisotropic functionality show the greatest potential for such 3D-printed hierarchical materials; for such materials, 3D printing can be leveraged to direct the self-assembly of these nanostructures in three dimensions, allowing “top-down” fabrication to meet “bottom-up” self-assembly. Such materials include liquid crystalline polymers and elastomers, as well as microphase-separated block copolymers.^8–11^ Here, we focus our attention on block copolymers as a chemically and functionally diverse class of materials characterized by their ability to readily form a rich array of ordered nanostructures; these self-assembling materials possess significant unexplored potential for multiscale 3DP-architected materials.^12^

To date, block copolymers (BCPs) have played diverse roles in additive manufacturing (AM) techniques, including as inks, additives to improve rheological or mechanical properties, and as support matrices.^12^ However, to date, only a select few 3D printing processes have extended beyond rheological applications to leverage the inherent self-assembly of BCP nanostructures to achieve truly hierarchical structural control.^13–17^ We have recently demonstrated the application of AM toward macro-scale (cm+) hierarchically structured block copolymers; using commercial thermoplastic elastomers, we fabricated simple mechanical metamaterials with tailored local mechanical anisotropy.^18^ However, the potential of nanostructured BCPs within additive manufacturing is still largely underexplored. Many existing and emerging additive manufacturing techniques have the potential to incorporate mechanisms to induce nanostructure orientation, in addition to existing geometric programmability, in order to facilitate controlled 3D architected BCP materials with locally tunable functional properties.^12^

Toward this goal, a wide range of mechanical or external fields can act as sources of BCP nanostructure alignment in advanced AM techniques. Extensive studies have demonstrated BCP orientation and established fundamental mechanisms governing orientation in response to both flow (shear^19–28^ and/or extensional^27–30^ ) and external fields (electric,^31–35^ magnetic,^36–41^ optical^42–45^ ). While there is potential to apply external fields as a source of orientation in additive manufacturing processes, here we focus on flow-induced orientation due to the compatibility with rapid “bulk” rather than thin “film” processing, as well as the inherent presence of flow in existing extrusion-based AM. In developing this nanostructure-informed AM approach, we take inspiration and insight from the extensive literature on flow-induced BCP nanostructure orientation. Decades of work have interrogated how the type and conditions (rates, thermal history) of the applied flow facilitate nanostructure orientation, investigating the impacts of key molecular factors including chain architecture, mechanical contrast, and the effects of “bridging” vs “looping” chain conformations in the development of proposed mechanisms.^22–25,46–60^ However, these studies have typically focused on relatively simple rheometric flows such as steady or oscillatory shear and uniaxial or planar extension, typically in isothermal environments and slow or long-lasting applied flows; all of these conditions represent major simplifications compared to the realities of melt extrusion-based 3D printing.

One distinct characteristic of the flow applied during 3DP is the presence of significant shear and extensional components within the nozzle, given the widespread use of convergent conical nozzles in the 3DP industry. In this geometry, both the flow type (shear vs extension) and flow rate vary spatially in both the radial and axial dimensions of the nozzle, leading to a complex, spatially heterogeneous flow history being applied to ink as it travels through the nozzle.^10,61^ A further source of complexity in 3DP of microphase-separated block copolymers is the complex flow-structure coupling due to the rheological impacts of flow-induced chain stretching and deviations in nanostructure order and orientation. As a result, there are no microconstitutive models for block copolymers that can comprehensively describe the relationship between a complex flow history, such as that applied in 3D printing, and the resulting state of the nanostructure and functional anisotropy upon 3D printing. However, techniques are being developed toward creating data-driven models to understand flow-structure coupling of complex fluids in mixed flow fields. One such example is the use of a fluidic four-roll mill (FFoRM) coupled with in situ spatially resolved SAXS measurements to train a machine learning model based on neural ordinary differential equations on the underlying flow-structure coupling.^62,63^ While such emerging techniques represent promising data-driven advancements in quantifying truly complex flows, they are currently limited to 2D geometries and are not yet implementable for high-temperature or high-effective-viscosity fluids, such as BCP melts. So, in extending the established ideas of BCP flow alignment toward rapid, modern AM techniques, we must interrogate ex situ how the spatially heterogeneous, nonequilibrium flow applied during printing (and following post-processing steps such as thermal annealing) impacts the final state of the BCP order and orientation.

Spatial heterogeneities in material structure resulting from a nonuniform AM flow history have been characterized in a variety of micro- or nanostructured 3DP inks, where subfilament scale structural variation provides insight into material orientation as well as local relaxation mechanisms. For example, microbeam scattering identified that an extruded clay/epoxy composite system exhibited flow-induced orientation particularly in close proximity to high-shear regions near filament walls and interfaces.^64^ Similarly, microbeam scattering has identified core–shell variations in the extent of mesogen alignment in 3D-printed liquid crystalline oligomers and polymers, both as a direct result of the flow rate in printing of liquid crystal elastomer precursors toward soft robotic LCEs,^10^ and as a result of rapid filament cooling “trapping” a highly oriented shell in a liquid crystalline polymer.^65^ Finally, in situ microbeam characterization of nanostructured block copolymer solutions showed subfilament heterogeneities in the direction of nanostructure alignment, including an apparent spatial flipping between lamellar alignment parallel and transverse to the filament axis.^66^ Based on these observations of subfilament heterogeneity in several extrusion-printed nanostructured materials, we anticipated that the mixed shear and extensional flow history applied to block copolymer melts may also lead to a distribution of nanostructure order and orientation.

Our prior work^18^ introduced a HOT-DIW 3D printing technique applied to a styrenic cylinder-forming triblock copolymer, showing that the flow applied in 3DP induces global nanostructure alignment directed along the printing path; however, several key mechanistic questions emerged. We found that printing rate and thermal annealing led to significant differences in long-range order, anisotropy, and heterogeneity. However, the filament-scale characterization performed on these materials did not enable detailed investigation of these local flow history variations, which left the underlying nature of some post-annealing variations in mechanical anisotropy unexplained.

Here, we apply scanning microbeam small-angle X-ray scattering (SM-SAXS) to probe the flow-induced spatial heterogeneities in nanostructure order and orientation in 3D-printed thermoplastic elastomer filaments. We characterize the heterogeneity in both the extent and direction of nanostructure orientation within 3D-printed filaments, both before and after a post-printing thermal annealing treatment. This characterization reveals the emergence of systematically off-axis tilted domains upon thermal annealing. The flow rate dependence of these tilted domains informs a proposed mechanism governing their emergence; namely, relaxation of asymmetric trapped stress from the 3D printing flow history promotes rotation-inducing torque on short, fragmented PS domains. Ultimately, we show that the presence and extent of off-axis tilting of BCP domains relative to the print direction is the source of the previously unexplained filament stiffness variations for certain flow conditions. These insights, which are uniquely accessible via microbeam X-ray scattering, highlight the capability of high rates of *combined* shear and extensional flow to lead to BCP domain misalignment as a result of extrusion-based processing. Insights such as these are critical to understanding how real processing flows impact the resultant structure and functional properties of materials derived from complex fluids such as the block copolymers studied here.

## Experimental Section

### Materials and Sample Fabrication

The triblock copolymer “G1648” (previously referred to as “MD1648” in our prior work) was provided by Kraton and was used as received. Material characterization and conditions for 3D printing and thermal annealing of this triblock polystyrene-*b*-poly­(ethylene-*co*-butylene)-*b*-polystyrene material were presented in our prior work and were followed exactly here.^18^ Briefly, this material has a total number-averaged molecular weight *M*_n_ = 43.6 kg/mol, *Đ* = 1.02, and is comprised of 19.4% polystyrene by weight, with glass transition temperatures identified as *T*_g,PS_ = 62 °C, *T*_g,PEB_ = −45 °C. This material has an equilibrium domain spacing of 20 nm between (10) planes, and retains the hexagonally packed cylinder geometry up to the order–disorder transition temperature of 190 °C, which is not exceeded in any processing presented here. For the printing conditions studied here, we quantify two key controlled process parameters: the average material velocity exiting the nozzle (*v*_avg_) quantifies the extrusion rate within the nozzle, and the draw ratio (*D*_R_) quantifies post-nozzle extension. *v*_avg_ is varied by increasing the extrusion rate (controlled by advancing a plunger through an ink reservoir via a stepper motor) in tandem with the nozzle translation rate, and can be understood as the velocity at which a filament would be extruded if the filament diameter matches the exit diameter of the nozzle. Post-nozzle extension, quantified by *D*_R_, is controlled by increasing the nozzle velocity (*v*_nozzle_) relative to *v*_avg_, related by the relation *D*_R_ = *v*_nozzle_/*v*_avg_.

### SM-SAXS: Nanostructural Characterization

SM-SAXS for top-down and end-on orientations was performed under vacuum at the 12-ID beamline at Brookhaven National Laboratory using a microfocused (2 μm × 25 μm) 14.5 keV X-ray beam using 0.5 s collection times. Line profile scans were performed across individual printed filaments in the “top-down” orientation with a scan spacing of 5 μm. 2-D area mapping scans were performed across thin (<1 mm) cross sections of filaments in an “end-on” orientation with 25 μm × 25 μm spacing.

Processing and reduction of these data was performed using custom Python scripts and the SMI beamline package. Background subtraction was performed on each scan to account for instrumental and environmental background. The spatially varied 2D scattering intensity distribution *I*(*q*,χ,*x*) was reduced to two 2D formats for visualization and analysis. *I*(*q*,*x*) is generated by performing an azimuthal integration across all data. The q-range describing the primary scattering peak (0.025–0.04 Å^–1^) was identified, and this q-range was radially integrated from *I*(*q*,χ,*x*) to obtain *I*(χ,*x*). For all 1D and 2D visualizations of spatial variation within a filament, a scattering intensity cutoff was implemented to distinguish scans containing sample from “empty” scans taken on either side of the filament. From *I*(χ,*x*), orientational order parameters ⟨*P*_2_⟩ (for all scans) and *C*_6_ (for end-on scans) were calculated following literature precedent, restricted to the q-range describing the primary scattering peak. Equation 1 shows the definition of ⟨*P*_2_⟩, which is the Hermans orientational order parameter, derived from the second-order Legendre polynomial describing the nanostructure orientation distribution.^67–69^ Equation 2 shows the definition of *C*_6_, which is a 6-fold bond-orientational order parameter, obtained via a Fourier series decomposition of the nanostructure orientation distribution.^70,71^

1 $$\left\langle P_{2}\right\rangle =\frac{3\left\langle \cos^{2}\chi \right\rangle -1}{2}$$

2 $$I\left(\chi \right)=I_{0}\left[\frac{1}{2}+\sum_{n=1}^{\infty }C_{n}\cos\left(n\chi +\chi_{n}\right)\right]$$

The position of all peaks of sufficient prominence in the *I*(χ) distribution for each scan was automatically identified in order to track the positions of all distinguishable populations of tilted domains. All values extracted from SAXS analyses that represent overall filament characteristics are the average taken from 3 replicate samples. All 1-D visualizations of scattering heterogeneity within individual filaments are one representative sample taken from triplicate measurements.

Additional SM-SAXS characterization in the “side-on” orientation was performed in an air environment at the Functional Materials Beamline (FMB) at the Cornell High Energy Synchrotron Source (CHESS). These SM-SAXS experiments were performed using a 2 μm × 20 μm, 9.7 keV X-ray beam with 0.1 s collection times and a Pilatus 300k detector. Line profile scans were carried out with 10 μm spatial resolution. Reduction of these data was performed using standard FMB beamline analysis workflows; analysis of reduced data was performed using analogous Python scripts and methods as described above for processing “top-down” scans from beamline 12-ID.

### Tomographic Deconvolution

Onion-peeling radial scattering deconvolutions were performed to extract “true” radial tilt profiles from inherently radially convoluted SM-SAXS data. To perform this deconvolution, the resolution was first reduced from approximately 80 scans to 21 segments (by binning and summing scans locally to mitigate error propagation^72^) spanning between *x* = 0 and *R*_fil_. For this analysis, we allow for radial variations of both the domain tilting profile and the extent of orientation, assuming both of these factors vary only as a function of radius. In the deconvolution, the outermost shell is analyzed to determine the local domain tilt at the location that is not convoluted with data from any other shells. Based on the best-fit value for the local domain tilt, scattering intensity scalar, and fwhm of the Gaussian-fit peak in *I*(χ) to capture orientation distribution, the predicted contributions of this shell’s scattering to all inner shells were subtracted. Following this subtraction, the next shell, one step toward the center of the filament, is no longer convoluted with outer shells and is amenable to repetition of the same procedure.

### SM-SAXS Simulations

To obtain the previously mentioned “predicted contributions” of outer tilted shells to the observed scattering throughout a filament, computer simulations of the contributions of each shell were performed by implementing the forward Abel Transform.^72^

3 $$I\left(\chi ,x\right)=\int_{-\infty }^{+\infty }F\left(x^{2}+y^{2}\right)dy$$

Briefly, integration of the expected observed scattering field *F* at each point along a vertical path along the “*y*” direction through a simulated filament yields a prediction of the local scattering pattern. By implementing this simulation at all spatial coordinates *x* within the sample, a complete simulation of SM-SAXS results is straightforward to obtain for any defined measurable scattering field in the sample. Schematics of this procedure, applied to an entire filament with a predefined radial tilt distribution, can be found in Figure S1.

When simulating scattering from axially tilted domains, the measurable scattering field is distinct from the field of true domain tilt angles, according to eq 4. Figure S2 shows a schematic derivation of this relationship.

4 $$\Delta \chi_{\mathrm{obs}}=\arctan\left(\tan\left(\Delta \chi_{\mathrm{true}}\right)\times \cos\left(\theta \right)\right)$$

As a result, the relevant observed field in eq 3 is Δχ_obs_ rather than Δχ itself, leading to suppression of the observed tilt for measurements taken near the “core” of such a cylindrically symmetric object. The suppression effect is visualized in Figure S3.

### Mechanical Characterization

Tensile testing was performed on an Instron 5965 equipped with a 500 N load cell. Single-filament samples for mechanical analysis were mounted in epoxy pucks with cyanoacrylate adhesive. Single-filament sample gauge lengths varied between 20 and 40 mm. During all tensile testing, strain was applied at 100% min^–1^ to a maximum strain of 100%. Printed filament diameters were measured directly with a micrometer and used to calculate a filament cross-section. Reported small strain modulus values were extracted from the small strain (0–2%) region and represent averages and standard deviations over 3–5 replicates.

### Rheological Characterization

For the investigation of stress relaxation, steady shear experiments were conducted using an Anton Paar MCR 501 rheometer with a cone and plate fixture (diameter 25 mm, cone angle 2°, truncation 50 μm). Shearing and relaxation steps were performed at the nozzle printing temperature (*T* = 160 °C), and samples were thermally disordered at 195 °C in the measurement apparatus prior to each measurement to minimize the effects of prior flow history.

## Results and Discussion

First, we outline the spatial heterogeneity expected of the flow applied within the convergent conical nozzle geometry, which is widespread in 3D printing. The internal flow profile is comprised of shear-dominated flow near the nozzle “walls” and extension-dominated flow near the “core” or central axis of the nozzle. In rheologically simpler inks, the relevant effects of the shear and extensional flow history can be adequately quantified using established constitutive equations and rheological modeling.^73–77^ However, when considering a nanostructured BCP melt in which the rheological properties and nanostructural order and orientation are deeply interconnected, it remains exceedingly difficult to quantitatively predict the true flow profile within a mixed-flow geometry. As such, we currently take a largely qualitative approach in discussing the flow history of these printed BCPs; we consider that filament “edges” are largely dominated by shear flow, the filament “core” is dominated by extensional flow, and that intermediate regions must experience intermediate, combined flow histories. A complete description of the local flow history would require extensive shear and extensional rheological characterization, which is out of the scope of this work. However, application of a few key assumptions (fully developed flow of a power law fluid with no wall slip) allows for estimated magnitudes of the expected shear and extension rates at the nozzle wall and along the nozzle centerline, respectively. These deformation magnitudes are shown in Figure S4 for the minimum and maximum printing rates explored here, along with the associated derivation. In addition to the radially dependent flow applied within the nozzle, DIW 3D printing typically requires the extruded material to undergo a 90° turn immediately upon exiting the nozzle; this imparts an additional “top-bottom” spatial heterogeneity in flow history. This aspect of the flow history has been shown both theoretically and experimentally to impact polymer entanglement and crystallinity in 3D-printed thermoplastics;^76–79^ we will address this aspect of the flow due to its potential nanostructural impacts. Finally, the rates of the applied shear and extensional flow also increase as the geometry converges. Investigation of the effect of nozzle design to tune the relative locations and magnitudes of the applied shear and extension is ongoing, but the analysis presented here focuses on one fixed nozzle geometry (Figure 1a) and does not address this aspect of the flow history. In this work, we will address spatial heterogeneities in 3D-printed BCPs which result both from radially varied flow inside the nozzle and the characteristic flow associated with this 90° turn.

**Figure 1:**
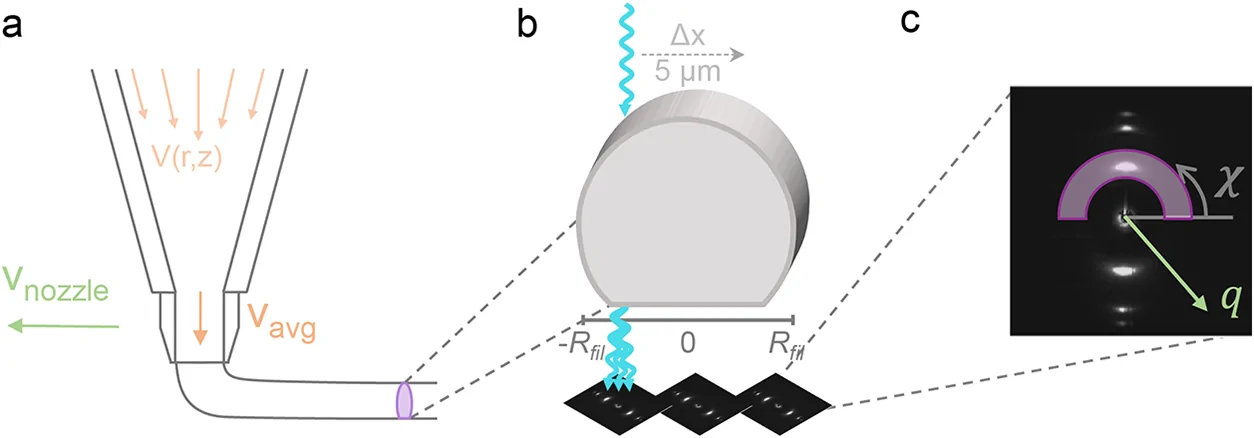
Sample fabrication and “top-down” SM-SAXS characterization configuration. (a) Schematic of 3D printing nozzle, where flow history upon printing is controlled by independently varying nozzle velocity (*v*_nozzle_) and average material velocity upon extrusion (*v*_avg_). (b) Schematic of sample configuration and data collection approach for “top-down” SM-SAXS collections. (c) For each scattering pattern collected, data is reduced as a function of scattering vector q and as a function of azimuthal angle χ, integrated across the q-range defining the primary scattering peak.

### Local Structural Characterization via Scanning Microbeam Small-Angle X-ray Scattering

Structural impacts from this spatially nonuniform flow are likely widespread in 3D-printed materials with complex nano- or microscale structures, but are difficult to characterize, typically requiring specialized techniques and/or extensive sample preparation. Here, we approach this characterization challenge by implementing a localized (2 μm × ∼20 μm beam) characterization technique, scanning microbeam small-angle X-ray scattering (SM-SAXS), shown in Figure 1. Using SM-SAXS, we characterize the subfilament structural heterogeneity in individual 3D-printed block copolymer filaments. This characterization was performed “ex-situ” at beamline 12-ID at Brookhaven National Laboratories, with additional measurements performed at the Functional Materials Beamline^80^ at the Cornell High Energy Synchrotron Source (CHESS). The approaches taken in all SM-SAXS experiments were similar; a microfocused X-ray beam was used to collect 2D-SAXS patterns with sufficient spatial resolution in order to resolve structural heterogeneities within the 400–700 μm diameter filaments. The majority of measurements presented derive from 1-D “line scans” with the beam in a “top-down” configuration, as shown in Figure 1b. Due to the transmission geometry of a beam passing through a filament with radially varying structure, data collected at any spatial coordinate “±*x*” (*x* = 0 @ filament center) will include convoluted signal from all radial shells within the range |*x*| < *r* < *R*_fil_. Analyses presented later in this work will address this deconvolution problem, but such deconvolution from a 1-D spatial scan requires the assumption of fiber symmetry. Our system is expected to deviate somewhat from this symmetry due to both the “bend” during printing and the flat bottom surface of the filament. As such, we primarily present the raw data without applying such deconvolution methods.

The 3-dimensional set of scattering data obtained from SM-SAXS “line scans”, *I*(*q*,χ,*x*), can be reduced to two 2D formats for visualization and analysis. First, *I*(*q*,*x*) can be generated by integrating each scan azimuthally, allowing for analysis of the extent of long-range nanostructure ordering, as well as any variations in domain spacing throughout the filament. From *I*(*q*,*x*), we can evaluate the heterogeneity in the peak center (q*) and full width at half-maximum (fwhm) of the primary scattering peak describing the hexagonal lattice of PS cylinders; these show minimal heterogeneity. Non-annealed filament cores show mildly (<5%) lower *q* peak centers in comparison to non-annealed filament edges or annealed filaments. From *I*(*q*,*x*), we also identify the *q*-range describing this scattering peak as 0.025–0.04 Å^–1^. The scattering intensity is then integrated across this q-range and plotted for each scan as a function of azimuthal angle χ to obtain our primary mode of data visualization: *I*(χ,*x*) for the primary scattering peak, shown in Figure 2a. This 2D reduction of scattering data allows for analysis of the extent of nanostructure orientation as well as any variations in the direction of nanostructure orientation relative to the filament axis; this is the basis for the majority of our analyses. An example of such data and the associated reductions for a 3D-printed filament is shown in Figure 2. We identify the azimuthal center of any peaks (χ_peak_) (Figure 2b) in the *I*(χ) distribution for each scan to track any deviation between the direction of nanostructure orientation and the filament axis (χ_0_), yielding the quantity Δχ = χ_peak_ – χ_0_ to describe any significant misalignment between the cylindrical nanostructure and the print direction. In Figure 2c, these azimuthal peaks are shown as a function of spatial position within a sample, with color intensity scaled by the relative peak intensities to visually indicate “smaller” peaks. We also calculate the extent of apparent unidirectional alignment via the orientational order parameter ⟨P_2_⟩; the radial heterogeneity in both ⟨P_2_⟩ and Δχ is shown in Figure 2c.

**Figure 2:**
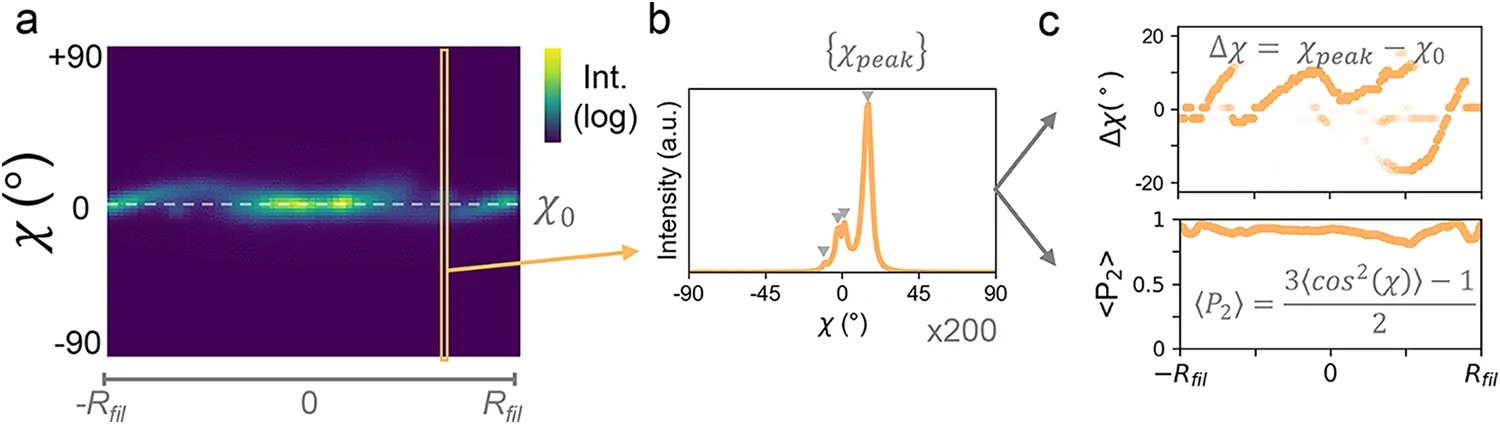
SM-SAXS line scan data analysis methodology. (a) 2D map of the azimuthal intensity distribution observed throughout one filament, *I*(χ,*x*), shown for the printing condition *v*_avg_ = 7.5 mm/s, *D*_R_ = 1. The dotted line labeled χ_0_ indicates the angular position of the scattering expected from domains which are oriented precisely along the filament axis. (b) For each scan’s azimuthal intensity distribution *I*(χ), all peak positions between −90 and +90° with sufficient prominence are identified as χ_peak_, representing the angular orientation of each distinguishable population of domains. (c) Reduced quantities as a function of scan position, calculated from *I*(χ,*x*). Δχ (top) shows deviation between the dominant direction(s) of nanostructure orientation compared to χ_0_, and the orientational order parameter ⟨P_2_⟩ (bottom) quantifies the extent of uniaxial alignment.

### Apparent Core–Shell Orientation Profiles Emerge upon 3D Printing

We first apply this SM-SAXS characterization approach to 3D-printed filaments which have not yet undergone post-printing thermal annealing; this analysis reveals the emergence of flow-rate-dependent “core–shell” profiles observed in the calculated order parameter ⟨P_2_⟩, shown in Figure 3. This observation confirms that the heterogeneous flow profile applied in 3D printing indeed leads to some degree of structural heterogeneity in printed filaments. Two aspects of the flow-rate dependence of this data are of most importance. First, these core–shell profiles are characterized by higher-alignment shells and lower alignment cores for samples printed above a critical average material velocity (*v*_c_ = 0.025 mm/s), which is consistent with the critical rate identified in our prior work. This printing rate was previously noted because it divides conditions based on the extent of mechanical anisotropy and nanostructure relaxation processes which are observed upon thermal annealing.

**Figure 3:**
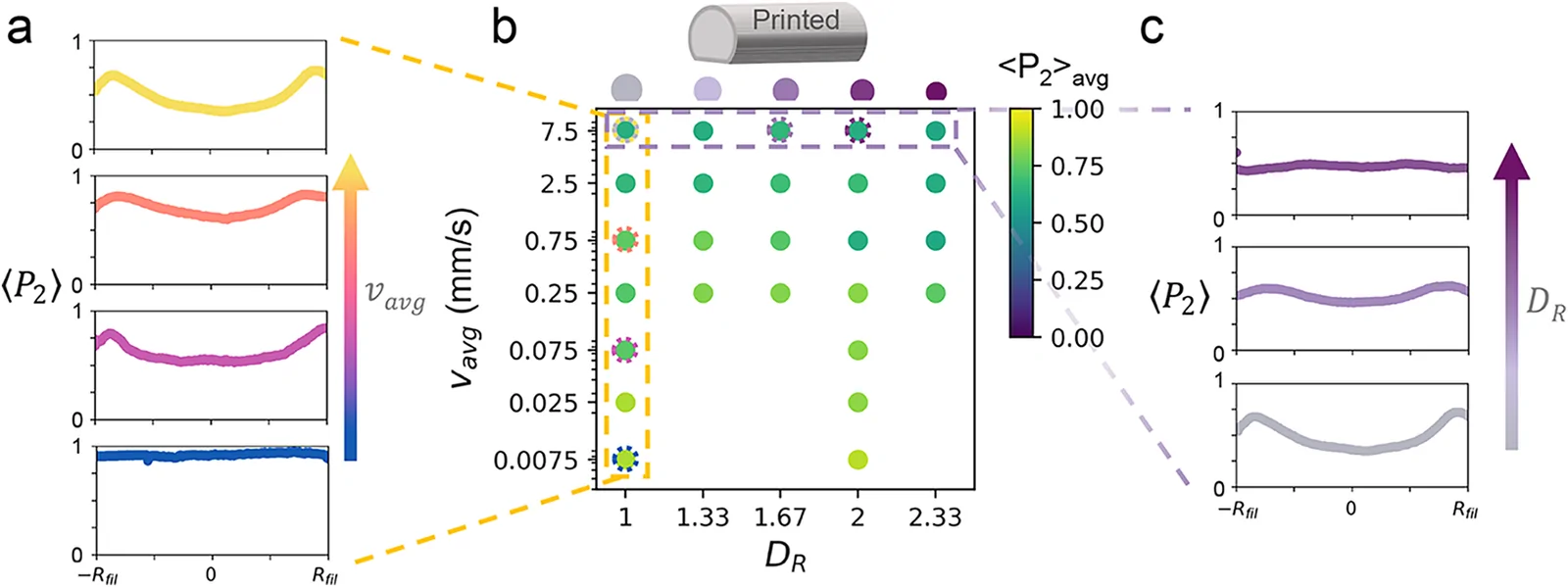
Emergence of core–shell nanostructure orientation profiles in printed SEBS filaments prior to thermal annealing. (a) Apparent radial orientation profile of a series of filaments printed at varying rates, with fixed *D*_R_ = 1, showing the emergence of a core–shell profile with a less oriented “core” in samples printed above a critical printing rate (*v*_avg_ = 0.025 mm/s). (b) Grid of printing conditions, with color representing the average “overall” value of ⟨P_2_⟩ for filaments printed at each condition. Dotted outlines indicate conditions highlighted in (a, c). (c) Apparent radial orientation profile of a series of filaments printed at varying *D*_R_ with fixed *v*_avg_, showing the suppression of the core–shell effect at *D*_R_ > 1.

The second feature of note is that the core–shell profile is suppressed at high draw ratio + high flow rate conditions in favor of spatially uniform “low” (0.5–0.7) values of ⟨P_2_⟩.

Altogether, the dependence of ⟨P_2_⟩ on flow rate and flow type provides insight into the distinct ways in which the shear- vs extension-dominated flows impact the underlying BCP nanostructure. It appears that high rates of extensional flow, observed locally in high printing rate filament cores and uniformly in high printing rate high *D*_R_ samples, tend to result in a suppressed ⟨P_2_⟩ upon printing. From prior TEM analysis, we know that such high printing rate samples exhibit a nanostructure characterized by short, discontinuous PS cylinders; this lack of long-range cylindrical continuity also results in lower nanostructural anisotropy.^81^ This phenomenon is illustrated through Fourier-transform analysis of TEM images of a high printing rate sample both before and after thermal annealing (Figure S5). Finally, from this characterization, we observe no major systematic tilting of nanostructure orientation in 3D printed filaments prior to annealing.

It is of interest to compare the polymer relaxation time scales with the observed critical rate which divides these distinct flow-structure regimes; doing so requires a quantitative estimate of the melt’s relaxation time. While ordinary linear homopolymer melts exhibit terminal relaxation times, relaxation in microphase-separated systems such as block copolymers is more complex; they lack a distinct terminal relaxation time, recovering it only when they cross through an order–disorder transition.^82^ Thus, they feature a microstructure-dependent relaxation spectrum; the microstructure (and thus relaxation processes) is dependent on the flow and deformation history. To estimate relaxation times, we were inspired by the approach of prior work that examined the relaxation dynamics of triblock copolymers following melt extension.^83^ We apply deformation at a given shear rate for a fixed total strain, measure the stress relaxation, and fit it to a multimode decaying exponential function to determine a spectrum of relaxation times (Figure S6); we report a weighted average relaxation time. Interestingly, we found that average relaxation times at 0.1 and 1 s^–1^ were 16 and 15 s, respectively. The relaxation spectra were likewise similar (see Table S1), ranging from the fastest subsecond relaxations to relaxation times of over a minute. We expect that higher-rate deformation will lead to faster relaxations, but experimental artifacts prevented data collection under these conditions. From these relaxation times, we can estimate shear Weissenberg numbers in the cylindrical exit of the nozzle for a few conditions: at *v*_avg_ = 0.0075 mm/s, we estimate *Wi* = 1.44, and for *v*_avg_ = 0.075 mm/s, we estimate *Wi* = 13.5. At the “critical” condition *v*_avg_ = 0.025 mm/s, and using an average relaxation time of 15.5 s, we find a “critical” *Wi* for the transition to long-range order of 4.7. Notably, these values are likely anomalously high as the relevant relaxation time is likely much shorter than the “average” relaxation time calculated from the multimode exponential fit. Fully evaluating the relaxation spectra would require truly mapping time-dependent relaxation of stress and microstructure evolution following deformation as a function of type, rate, and strain.

### Tilted Domains Develop Upon Thermal Annealing of 3D Printed Filaments

SM-SAXS of filaments which have been thermally annealed reveals a striking difference in the scattering signature compared to non-annealed filaments. For a similar set of flow conditions as those which lead to core–shell orientation profiles upon printing (high printing rate, low-moderate *D*_R_), subsequent thermal annealing leads to the development of off-axis tilted BCP domains (Figure 4), as quantified by local measurements of Δ*χ* well above 0°. Within the range of flow conditions which leads to these domain tilts, we observe an increase in the extent of tilting angle Δ*χ* with printing rate, reaching up to 20° at *v*_avg_ = 7.5 mm/s. The spatial-dependence of Δ*χ*, shown in Figure 4, indicates that the nanostructure is aligned along the print direction locally at the filament edges (*r* > 0.9 × *R*_fil_), but deviates in an intermediate radial range (*r* < 0.8–0.9 × *R*_fil_). Near the filament core, the nanostructure may be assumed to be aligned along the print direction due to the symmetry of the flow history, but SM-SAXS using only the primary “top-down” measurement configuration cannot directly provide validation of this. We note that in interpreting these observed Δ*χ* values as axial “tilts”, we assume the absence of any helical tilting behavior, which is justified here due to lack of mirror-symmetry breaking in the 3DP process. Finally, we note a few anomalously high values of Δ*χ* at a few high *D*_R_ conditions. In the highest *D*_R_ conditions (*D*_R_ = 2.33), the high amount of post-nozzle extension can lead to macroscale rheological instabilities (See Figure S7). These significantly alter the characteristic flow history and may lead to the anomalous high *D*_R_ results shown in Figure 4b. In the lowest printing rate condition for *D*_R_ = 2, we suspect that the proximity of these rheological instabilities as well as the susceptibility to minor thermal fluctuations on the time scale of printing may both influence the observed behavior.

**Figure 4:**
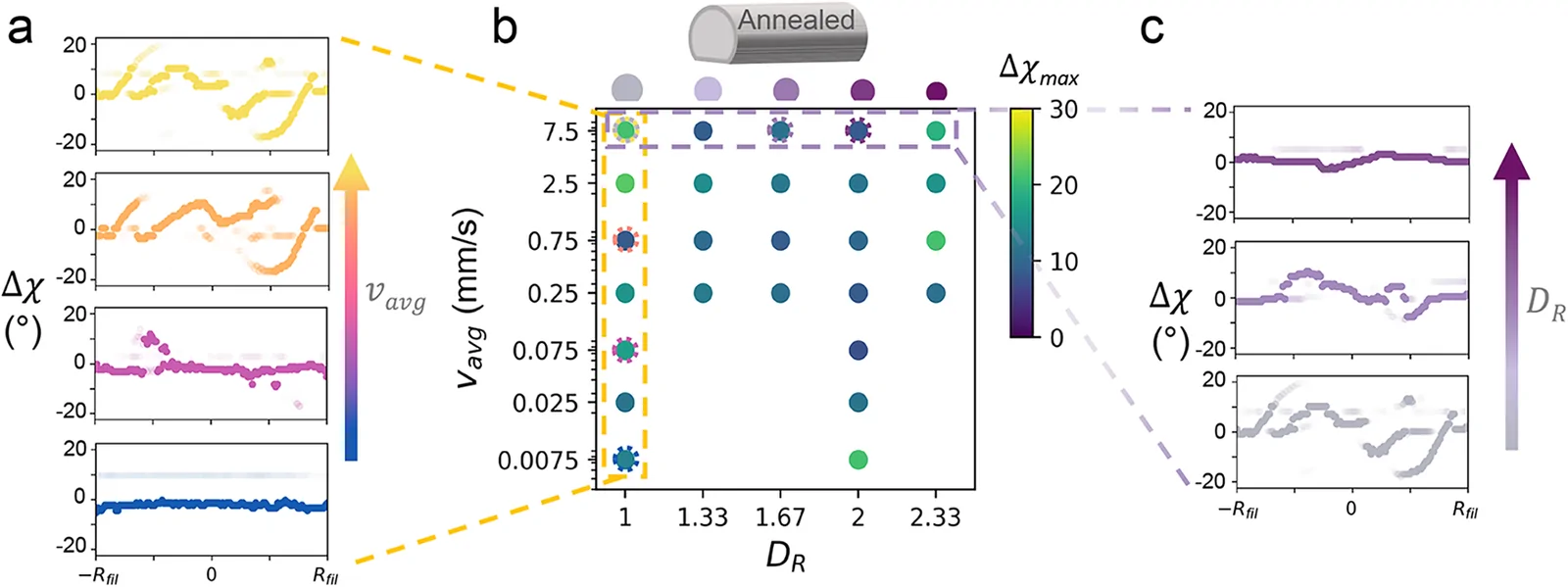
Tilted domains emerge after thermal annealing of printed SEBS filaments printed at select flow conditions. (a) Δχ of samples printed at varying rates with fixed *D*_R_ = 1. Populations of domains which are “tilted” with respect to the filament axis emerge in samples printed above a critical extrusion rate. (b) Grid of printing conditions, with color representing the maximum value of Δχ (weighted by each peaks’ intensity) for filaments printed at each condition. Dotted outlines indicate conditions highlighted in (a,c) (c) Δχ of samples printed at varying *D*_R_ with fixed *v*_avg_, showing the suppression of the “tilted” domains for flow conditions with *D*_R_ > 1.

Orthogonal SM-SAXS measurements in supplemental “side-on” and “end-on” configurations provide additional insights regarding how flow from 3DP impacts these tilted BCP nanostructures. Side-on measurements of select annealed samples which exhibit tilted BCP domains reveal a significant top-bottom asymmetry in the observed nanostructure tilting phenomenon (Figure 5). This asymmetry is presumed to result from the top-down asymmetric flow history as has been modeled and described for 3D printed polymer melts (See Figure 1).^76,77^ Further, these side-on measurements are consistent with our expectation that the nanostructure at the filament core is indeed aligned along the print direction. However, due to the still heavily convoluted scattering signal recorded in measurements of the “core” in either side-on or top-down orientations, this remains challenging to confirm without signal deconvolution. True validation of the nanostructure layout in the filament core, without interference from signal originating from outer layers, requires a third orthogonal measurement configuration.

**Figure 5:**
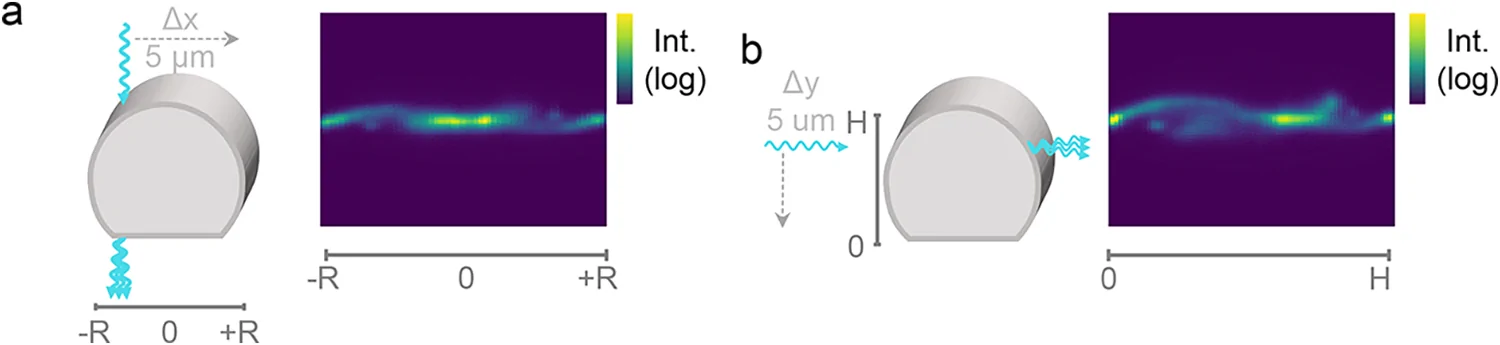
Asymmetry in radial domain tilting observed via side-on sample characterization, shown for the printing condition *v*_avg_ = 7.5 mm/s, *D*_R_ = 1. (a) Schematic of the sample configuration and associated antisymmetric I­(χ,x) from a “top-down” measurement indicating left/right symmetry. (b) Schematic of the sample configuration and associated asymmetric I­(χ,y) from a “side-on” measurement indicating top/bottom asymmetry.

SM-SAXS collections taken in an end-on configuration in which the X-ray beam is oriented parallel to the filament axis and rastered in a 2D grid across the filament uniquely provide a full 2D mapping within a thin (<1 mm) filament cross-section. This cross-section mapping approach has fundamental similarities to scanning probe TEM diffraction,^84^ but is suitable for the macroscopic sample dimensions which are convenient here. For measurements in this orientation, additional order parameters are necessary to analyze the extent and direction of nanostructure orientation. To visualize these 2D maps, we compute both 2-fold and 6-fold order parameters, ⟨P_2_⟩ and C_6_, respectively, shown in Figure 6.^70,71^ For a lattice of cylinders oriented along the X-ray beam path, the expected scattering pattern possesses 6-fold symmetry. If this lattice of cylinders is oriented at a considerable offset to the beam path, such as we know to exist in samples with axially tilted domains, four of these spots are suppressed, leading to a primarily 2-spot pattern oriented in a direction which can indicate the local direction of the tilt, as shown in Figure S8. An additional complication is that if measurement locations contain multiple grains within the sample thickness, the observed scattering can possess multiple overlapping 2-fold and/or 6-fold symmetries. With these complications in mind, quantitative interpretations of data from this end-on interpretation are challenging. However, qualitative radial effects are interpretable; results from a selected print condition both prior to and after thermal annealing are shown in Figure 6.

**Figure 6:**
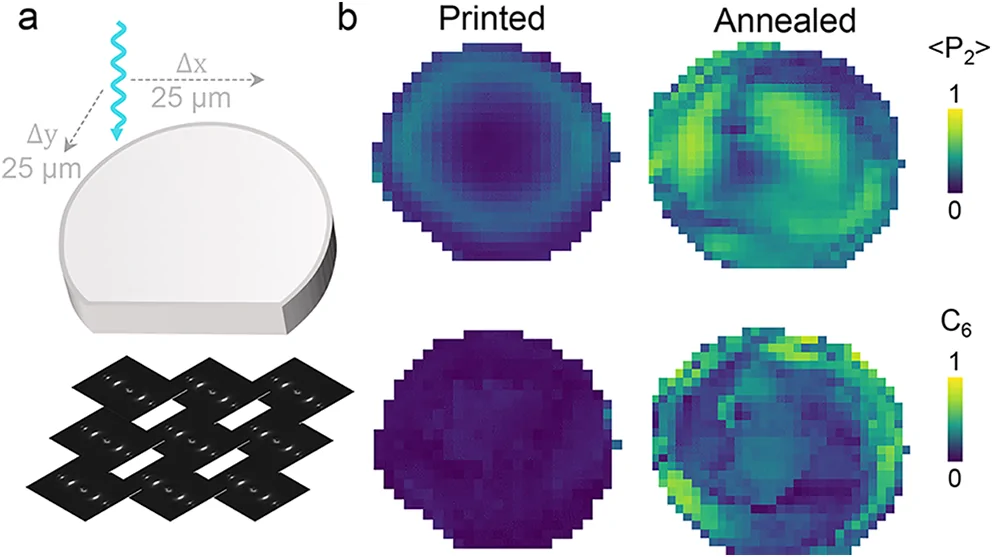
Radial dependence of domain tilting observed via “end-on” 2D-SM-SAXS characterization, shown for the printing condition *v*_avg_ = 7.5 mm/s, *D*_R_ = 1. (a) Schematic of microbeam scanning configuration for 2D-maps of filament cross sections. (b) Apparent 2-fold (top) and 6-fold (bottom) symmetry observed in cross sections of samples upon printing (left) and after subsequent thermal annealing (right).

The printed sample shows a minor degree of observable 2-fold symmetry near the outermost shell, indicating some minor presence of domain tilting in the outermost regions of the printed filament prior to annealing. The extent of apparent tilting observed here is sufficiently minor that it is not observable in “top-down” filament measurements. This sample exhibits minimal 6-fold symmetry, as expected based on prior analysis suggesting a lack of long-range hexagonal ordering upon printing. However, the annealed cross-section clearly shows 6-fold symmetry to be concentrated in an outer radial band and in the inner core of the filament, while 2-fold symmetry is dominant throughout, mostly at intermediate radii. The compatibility or overlap between regions with these computed order parameters is shown in Figure S8. Similar end-on characterization of a high *D*_R_ filament following thermal annealing is shown in Figure S9; this case shows a “global” presence of observed 2-fold symmetry in one particular direction. Given our strong understanding of the sample orientation from orthogonal measurements and the observed functional anisotropy, we are confident that this observed symmetry is representative of a macroscopic tilt of the sample relative to the beam, highlighting the high sensitivity of these measurements to sample mounting orientation. Future work would benefit from systematically tilting the sample stage to optimize sample orientation prior to this type of measurement (or even conducting such measurements at several tilt angles of the sample cross-section relative to the beam, to enable enhanced 3D reconstruction).

Taken together, the results from these three orthogonal characterization directions of high extrusion rate, low *D*_R_ samples shown in Figures 5 and 6 clearly elucidate a radially varied orientation profile in which cylinders are oriented along the filament axis at the outermost shell and the innermost core, while intermediate radial regions exhibit tilting directionally “toward” the filament axis. Based on the known orientation of the filament print direction, we also note that these tilt angles are pointed “inward,” or showing a similar direction of orientation to the angle of the sloped nozzle walls, rather than the opposite tilt direction which may result from “outward” expansion-type flows such as die swell. The hypothesized origin of this domain tilting behavior will be discussed later in this work.

### Tilted Domains Impact the Mechanical Anisotropy of 3D Printed Filaments

From these comprehensive multi-orientation SM-SAXS characterizations, we have confirmed the systematic development of off-axis tilted BCP domains in regions of printed filaments which experience a flow history characterized by high rates of in-nozzle shearing followed by in-nozzle extension. The resulting heterogeneous alignment direction within individual filaments has critical implications for the resulting functional anisotropy. In the SEBS studied here, our exploration of functional anisotropy to date has focused on small-strain mechanical properties. The angular-dependence of the small-strain modulus of a unidirectionally oriented cylindrical styrenic triblock copolymer has been established and thoroughly characterized; eq 5 defines this relationship.^85^

5 $$\frac{1}{E_{\theta }}=S_{11}\sin^{4}\theta +{{(2S}_{13}+S_{44})\sin}^{2}\;\theta \;\cos^{2}\theta +S_{33}\;\cos^{4}\theta$$

This relationship is defined by three components of the anisotropic stress tensor (S) for an assumed “single crystal” of oriented TPE. S_11_ and S_33_ are elastic compliance coefficients, where compliance is related to the inverse modulus. These 11 and 33 elements of the strain tensor represent the behavior along and perpendicular to the cylinder axis or print direction, and are determined from tensile deformation of sheets in each of these directions. S_13_ and S_44_ are elements of the strain tensor which reflect the material’s compressibility and shear compliance, respectively. These terms are determined in combination (2S_13_ + S_44_) from tensile deformation of a sheet at a 45° offset to the print direction. We have calculated these coefficients for our system using sheets fabricated at high *v*_avg_ and high *D*_R_ conditions. These conditions yield a nanostructure which is globally unidirectionally oriented with long-range domain continuity, and with little to no observable domain tilting, so we treat this as a near ideal “single-crystal” architecture. Figure S10 shows the small strain region of the stress–strain curves for these three orientations, from which E_0_, E_45_, and E_90_ were extracted. Fitting of these angular-dependent moduli following eq 5 yields the following coefficients: S_11_ = 0.6317, 2S_13_ + S_44_ = 0.64027, and S_33_ = 0.008026 (MPa^–1^). With eq 5 describing the angular-dependence of the small-strain modulus, we apply this toward a predictive model describing the mechanical impacts of tilted domains within 3D printed TPEs.

Following eq 5, the stiffness of our materials decays by a factor of 2 at only a 6.5° offset between the orientation direction and the measurement direction, and by a factor of 10 at a 20° offset. The presence of identifiable populations of domains from SM-SAXS which are tilted at angles up to 20° relative to the filament axis is expected to have a drastic impact on the resulting filament stiffness. Further, the stiffness of a filament is highly dependent on the precise extent of tilting as well as the relative volume fractions of tilted domains within a filament. In order to quantitatively verify this, we require both a suitable method for identifying the complete population of tilted domains within a filament, and a mechanical model to calculate an expected filament stiffness based on this population.

The simpler of these two problems is implementing a mechanical model to infer filament stiffness as a result of the population of tilted domains. Our prior work utilized an extension of the Takayanagi model for straining stiff and soft BCP domains either in parallel or in series, applied to a macroscopic composite defined by the programmed geometry imposed by the print path. Here, we apply the same composite treatment, considering an individual filament as a composite of multiple BCP “components” strained at varying directions relative to the cylinder orientation axis. As such, calculation of a filament stiffness is obtained by considering each of these components being exposed to strain in a parallel configuration. The resulting equation defining the filament stiffness is shown in eq 6.

6 $$\frac{1}{E_{\mathrm{pred}}}=\sum_{\Delta \chi =0}^{90}\rho \left(\Delta \chi \right)\times \frac{1}{E\left(\Delta \chi \right)}$$

With eq 5 describing *E*(Δχ), the required “input” to this equation is solely ρ­(Δχ), the set of coefficients (summing to 1) describing the relative populations of domains at any given tilt angle Δχ. In order to obtain this distribution from the SM-SAXS result, we must address the inherent signal convolution challenge.

### Tomographic Deconvolution Extracts Effective Radial Variations in Nanostructure Orientation

A complexity of the data collected in this work is the convolution of the measured signal at any location with signal originating from outer “shells” of the filament. Measurements taken near the center of the filament include scattering signal from every “shell” with a larger radius. In this work, we apply a relatively simple method of 1D tomographic deconvolution, termed the “onion-peeling” approach.^86^ The primary modification developed for our work stems from the need to account for the “tensor tomography” character;^87^ or to account for tilted domains which do not “uniformly” contribute to scattering at all measurement locations. For example, consider that an outermost radial band (*x* > 0.95 × *R*_fil_) may be comprised of nanostructures which are tilted Δχ° relative to the filament axis. When measured at *x* = 0.97 × *R*_fil_, this tilt is mostly in the measurement plane, and would be measured appropriately by the detector in the form of an angular offset of approximately Δχ° between the position of the scattering maximum in *I*(χ) relative to the filament orientation. However, assuming fiber symmetry, when this outer shell is measured at the position *x* = 0, the Δχ° tilt does not meet the necessary geometric condition to be observed in the measured signal; here signal would be observed without any angular offset representing this off-axis tilt, and may be subject to depressed intensity. Schematics visualizing these tilt-suppression effects are shown in Figure S4.

Briefly, incorporating this complexity in the onion-peeling style analysis requires identification of the structure in the outermost “shell” first, followed by a simulation of the predicted contributions from this shell to the measurements collected at each measurement location, considering the dependence of observed “tilt angle” as a function of measurement position. The predicted contributions of the outermost shell are then subtracted from the data. This process is repeated iteratively in order to identify the “true radial scattering” results. Due to the considerable simplifying assumption of fiber symmetry required for this analysis, we have so far presented the “raw” or “convoluted” visualizations of the data throughout this work. But, for the following analyses, we now find these deconvolution assumptions necessary in order to extract approximate radial variations and relative populations of tilted domains. While the number of scans collected across the width of a filament is near 100, error propagation in tomographic deconvolution compounds with the number of “shells” considered; as such, here we bin data radially into 10 regions for the deconvolution analysis.^72,86^ In the deconvolution analysis applied here, both the direction (Δχ) and extent (⟨P_2_⟩) of nanostructure orientation are permitted to vary within the filament; fitting the outer shell involves identifying the position and distribution of scattering intensity, allowing for monitoring of both relevant orientation properties.

From applying this deconvolution, we extract effective radial profiles of the extent and direction of nanostructure orientation, plotted in Figure 7 for a sample exhibiting tilted domains. The tilting is shown to be localized in intermediate radial regions, and the extracted value of ⟨P_2_⟩ remaining >0.9 at nearly all locations confirms a high degree of local unidirectional ordering, supporting the application of the single-crystal mechanical model to describe the stiffness of this system. Critically, we also obtain the population distribution of domain tilts present in the sample, shown for a representative sample in Figure 8b. From these extracted tilt populations, we can now generate nanostructure-informed predictions of mechanical properties for each printing condition, and compare with the measured mechanical properties, shown in Figure 8c. The prediction works reasonably well for samples printed above the critical printing rate previously identified, and clearly fails for a select few samples for which the model overpredicts the modulus. The samples for which the modulus was most greatly overpredicted were the two lowest printing rate samples, for which we would not expect this analysis to succeed; prior TEM and mechanical analysis suggested a well-oriented structure lacking long-range cylinder continuity, which is not accounted for by this mechanical model. Ultimately, the tilt population distributions extracted from tomographic deconvolution of these SM-SAXS measurements, though certainly not precisely quantitative due to the inherent assumptions of symmetry and long-range (≫1 μm) domain continuity,^81^ account well for the observed variations in actual functional anisotropy.

**Figure 7:**
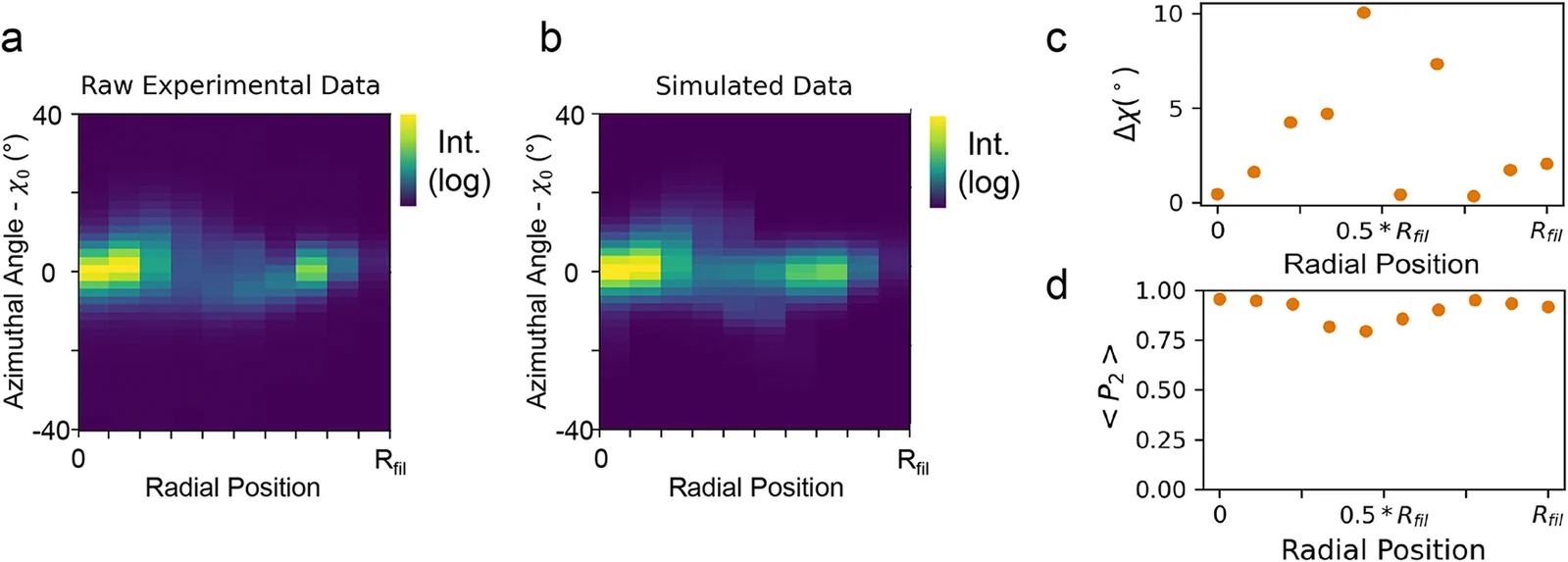
Results of tomographic deconvolution of SM-SAXS, shown for the printing condition *v*_avg_ = 7.5 mm/s, *D*_R_ = 1. (a) Experimental data, trimmed to include only one-half of the filament and spanning a narrower range of azimuthal angles. Measurements were further binned spatially to mitigate onion peeling error propagation. (b) Simulated data obtained from onion-peeling analysis of the data in (a). (c) Extracted radially dependent tilt profile from onion-peeling analysis, indicating minimal angular offset in the core and outer shell, with the majority of tilting attributed to intermediate radial bands within the filament. (d) Local measurements of orientational order parameter extracted from onion-peeling analysis, showing extremely high values of ⟨P_2_⟩ (>0.9) with localized deviation at an intermediate radial band near 0.4 × *R*_fil_. This local deviation may be stem from the region of filament-substrate contact at the bottom of the filament, where the inherent assumptions of fiber symmetry are likely oversimplifying.

**Figure 8:**
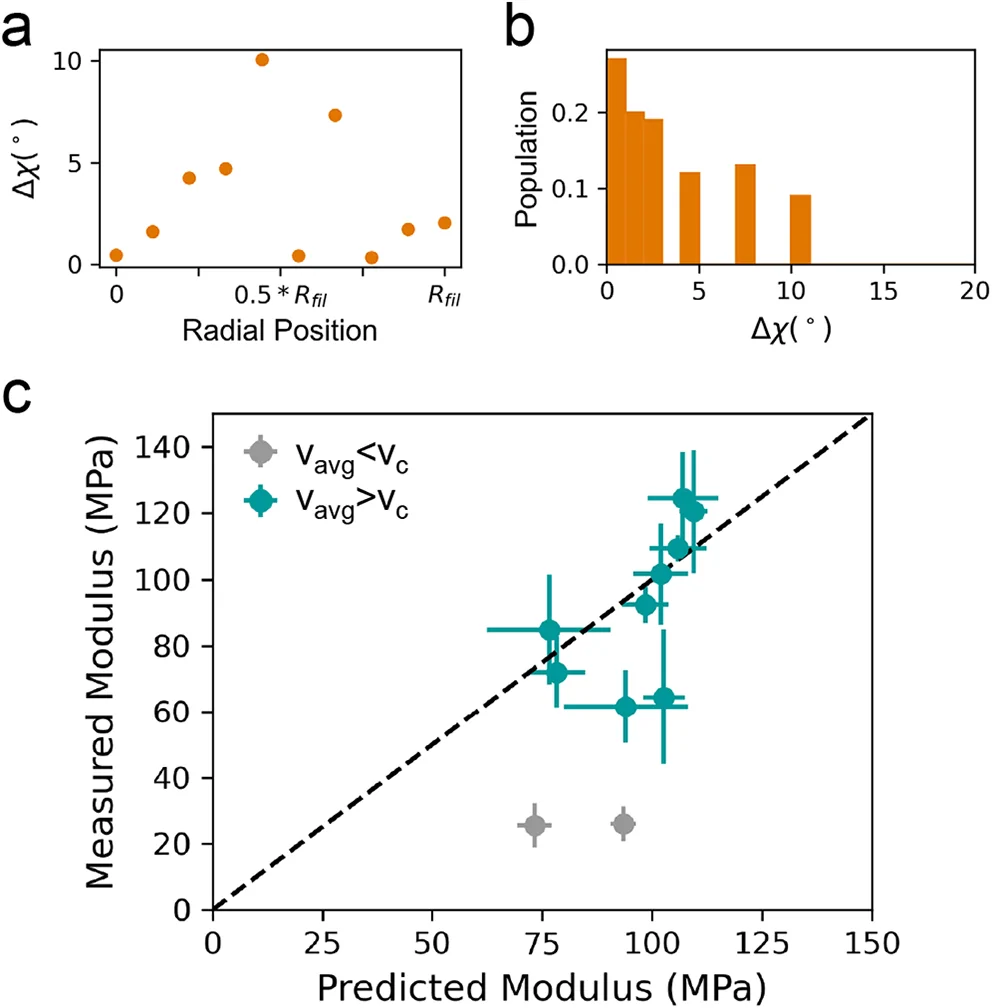
Population distribution of tilted domains governs mechanical anisotropy in annealed samples. (a) Δχ­(*r*) for the representative annealed filament discussed in Figure 7, obtained via onion-peeling analysis. (b) Relative populations of domains tilted by angle Δχ with respect to the filament axis, calculated by weighting the local extracted tilts from (a) by the relative area of each radial “shell” of the filament. (c) A reasonable agreement is observed between filament mechanical properties predicted via eq 6 and measured filament mechanical properties among this set of annealed samples. The two samples shown in gray were printed below the critical printing rate (*v*_c_ = 0.025 mm/s) discussed previously, preventing the formation of long-range domains.

This discussion has focused on tomographic deconvolutions of the annealed filaments which exhibit significant domain tilting behavior; filaments which have solely been printed may also be subjected to similar deconvolution processing to extract true “radial” profiles in the extent of orientation. Such processing reveals an easily predicted outcome; the slightly more highly oriented “shell” increases the “observed” extent of orientation near the filament core compared to the “true” extent of orientation near the core; similar insights have been discussed in core–shell 3DP-oriented LCEs.^10^

### Proposed Mechanisms Governing Emergence of Tilted Nanostructures

Next, we propose a series of mechanisms for how the flow applied in 3D printing leads to the observed ordering and orientation upon printing and thermal annealing, in particular the unique “tilted domains”. There are four distinct flow histories which we consider: (1) high rates of in-nozzle shearing, followed by in-nozzle extension, (2) high rates of “pure” extension, whether in-nozzle or external, (3) high rates of “pure” shear, and (4) low rates of shear and/or extension.

Scenario 1 (Figure 9a) describes a proposed flow deformation pathway which leads to the observed domain tilting upon thermal annealing. We imagine that first, BCP cylinders are aligned by shear in register with the angle of the nozzle wall (15°). This is followed by a transition to high extensional flow near the junction to the cylindrical exit region of the nozzle. The shear-alignment, when applied above a critical chain relaxation rate, results in asymmetric chain extension (Figure 9ai). When this stressed, sheared structure is further exposed to a critical level of extensional flow, chains are further stretched along the printing direction, resulting in cylinder-breakup, which we have characterized by TEM (Figure S6). This flow history is expected to result in chain extension with an off-axis tilt. For a short PS domain, the force experienced as these stretched, bridging BCP chains relax toward their equilibrium configuration would induce a net torque on each domain, driving reformation of PS cylinders at an angle which is dependent on the extent of trapped stress within the chains upon printing (Figure 9aii). We note that these tilted domains only appear in regions of the nozzle subject to this “mixed flow” containing both shear and extensional character, consistent with our proposed mechanism.

**Figure 9:**
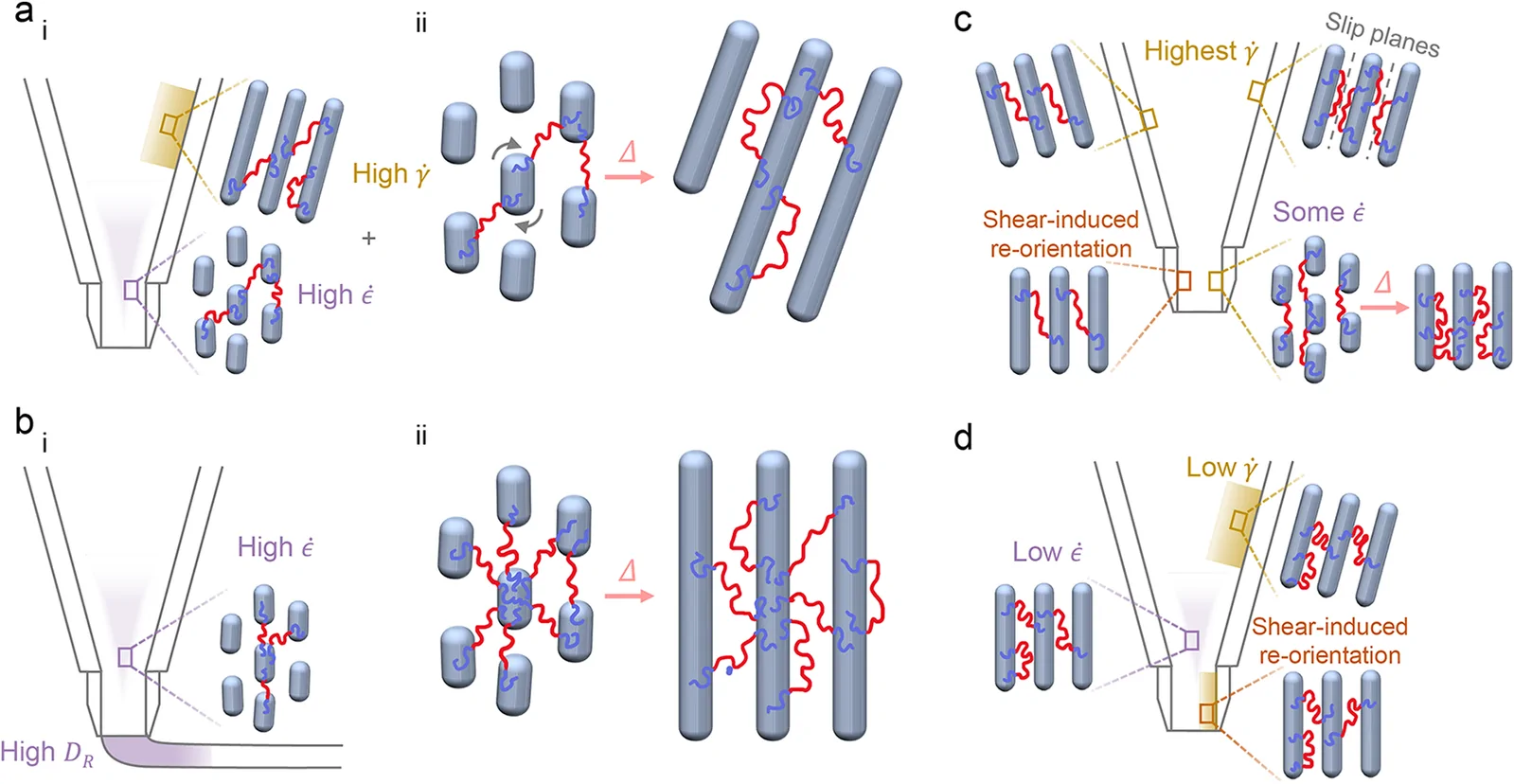
Proposed mechanisms governing domain tilting and nanostructure orientation in response to the mixed flow profile applied in extrusion 3D printing. Subparts a,b,c refer to structure development under high flow rates; subpart d refers to structure development at very low flow rates. (a) Shear followed by high extension (i) is proposed to lead to the formation of tilted domains after thermal annealing due to relaxation of asymmetric trapped chain stretching inducing torque on fragmented PS domains (ii). (b) Regions dominated by extension (i) lead to symmetric trapped stress which provides no driving force for domain tilting (ii). (c) The highest shear rates near nozzle walls may facilitate shear-induced reorientation within the cylindrical exit region (left) or the formation of slip planes may mitigate shear-induced asymmetry in trapped chain stretching. (d) Printing rates below a critical chain relaxation time scale threshold do not develop significant trapped chain stretching upon extrusion.

We note that die swell may be proposed as an alternative flow characteristic leading to the observed domain tilting phenomenon. However, the observed filament dimensions do not clearly vary with extrusion rate, as would be expected if die swell was the mechanism underlying the nanostructure tilting. Further, work by other groups leveraging in situ microbeam SAXS during extrusion of nanostructured (cylindrical and lamellar) poloxamer solutions observed a qualitatively similar nanostructure orientation direction following extrusion through a straight channel nozzle.^66^ The extent of poloxamer nanostructure tilting persisted across all measured solution compositions and nozzle dimensions, in systems both with and without observable die swell. Only one poloxamer lamellae-forming composition studied exhibited die swell; in this case, the rapid “expansion” associated with the die swell resulted in temporary observable nanostructure orientation in the opposite direction of the “persistent” orientation direction. The persistent orientation direction in the extruded poloxamer solutions is consistent with the direction of orientation in the TPE block copolymers melts used in our work. Ultimately, the independence of the geometrically observable die swell effect and the characteristic nanostructure tilting observed in both these TPE block copolymers and in the poloxamer solutions previously studied support that die swell is not driving this off-axis nanostructure orientation.

Scenario 2 (Figure 9b) describes the effects of near-pure high strain rate extensional flow at the filament core or in high *D*_R_ flow conditions. In this case, high strain rate extensional flow similarly results in trapped chain extension along the printing direction, but the symmetric, uniaxial stretching and resulting trapped stresses do not impose any rotation-inducing forces on the fragmented PS domains upon annealing (Figure 9bii). This symmetry allows for recovery of domain continuity along the direction of imposed extension. The uniaxial extensional flow applied across the entire filament in high *D*_R_ conditions progressively suppresses the degree to which domain tilting develops. Prior to the post-nozzle extension, chains are expected to possess the asymmetric stress described in Scenario 1; with sufficient extension to drive highly stretched chains’ end blocks to pull out and re-enter a different PS domain, the asymmetry in chain conformation can be to some degree “erased” in favor of a less asymmetric state of trapped stress, reducing the extent of observed domain tilting at high draw ratio conditions.

Scenario 3 of rapid “pure” shear addresses the behavior near filament edges, where the maximum shear rate is observed and where extensional flow components are minimized. Two proposed chain and nanostructure reorientation pathways are shown in Figure 9c. From SM-SAXS, these regions exhibit higher apparent anisotropy than the extension-dominated cores, and retain orientation along the print direction after thermal annealing. This “shell” region also exhibits a higher apparent degree of nanostructure orientation, suggesting the extension-induced domain breakup leading to suppression of ⟨P_2_⟩ is a less significant factor of the flow history within this region. One flow-mechanism which may underlie this behavior and cause the distinction between Scenarios 1 and 3 is the generation of effective slip planes, or a shear-induced transition toward a looping rather than bridging chain conformation. This “slip plane” formation has been proposed and documented in lamellar BCP melts in response to high shear strains, though it has not been quantitatively shown in cylinder-forming materials.^46,88^ If the high shear rates near the nozzle wall facilitate slip plane formation, this would drive the majority of stretched chains to originate from a looping conformation rather than a bridging conformation, therefore minimizing the driving force for domain recombination at an angle relative to the filament axis; this pathway is shown on the right of Figure 9c. Alternatively, the high rate of applied shear within the 1 mm cylindrical nozzle exit may be sufficient to reorient these domains to be in register with the “straight” channel wall, and the minimal extensional flow component adjacent to the walls may prevent significant PS cylinder breakup upon printing; this pathway is shown on the left in Figure 9c.

Scenario 4 uniquely results in printed filaments without significant trapped chain extension or domain breakup, shown in Figure 9d. This scenario is characterized by shear and/or extensional flow rates applied below a critical rate of chain relaxation. Here, any cylindrical domains or grains which lack long-range continuity upon 3D printing also lack a sufficient chain-level driving force to recover that continuity across a sufficient length-scale for stiff mechanics to emerge following annealing. We propose that this lack of long-range continuity may stem from grain boundaries which lack cylinder-continuity and which may persist through the extrusion process. Ultimately, this slow extrusion rate condition leads to the uniform observed morphology of well-ordered cylinders upon printing, as well as limited functional anisotropy even after thermal annealing. We note that these low printing rate conditions which lack mechanical anisotropy highlight a limitation of using X-ray characterization to predict functional properties which are dependent on the long-range continuity of the PS domains. Such low printing rate conditions are uniquely identifiable via SAXS of printed filaments based on the increased sharpness of the primary scattering peak; the fwhm of this peak as a function of printing and annealing conditions is shown in Figure S11 However, this scattering feature only provides a correlation between an aspect of the printed nanostructure and the resultant post-printing mechanical anisotropy in this material, rather than providing a direct, quantifiable measurement of the relevant structural feature. Such long-range domain continuity characterization would require an extensive tomographic TEM approach or application of slice-and-view SEM to probe continuity over the required length scale of several microns, highlighting the significant challenges in achieving comprehensive structural characterization of complex 3D nanostructured materials.

## Conclusion

In this work, we have leveraged SM-SAXS as a powerful local nanostructure characterization method toward understanding property-relevant subfilament structural heterogeneities in 3D printed thermoplastic elastomer triblock copolymers. Local structural measurements revealed that upon thermal annealing, well-ordered cylindrical domains develop which are tilted toward the filament axis at angles of up to 20°, dependent on the flow conditions of fabrication. These tilted domains emerge only in intermediate radial “bands” for undrawn filaments printed at high deformation rates, indicating that the flow history which leads to this behavior is the distinct combination of select rates of in-nozzle shearing followed by moderate in-nozzle or post-nozzle extension. Following these structural insights, we presented a proposed mechanism by which asymmetrically stretched bridging chains drive reformation of cylinders at an angular offset to the filament axis. Further, tomographic deconvolution assuming uniaxial symmetry of these SM-SAXS yielded a population distribution of domain tilts which was used to generate predicted Young’s modulus values for each flow condition; these predictions showed reasonable agreement with experimentally measured mechanical properties for all samples exhibiting long-range cylinder continuity. The subfilament heterogeneities elucidated here highlight the importance of precise characterization to identify how complex AM flow histories interact with anisotropic, structured fluids to guide the resultant functional material properties.

## Supplementary Material



## Data Availability

All data relating to this publication are openly available from Princeton DataSpace (DOI: https://doi.org/10.34770/14zc-5c22).

## Notes

Visualized procedure for simulated SM-SAXS collections, derivation and impact of nanostructure tilt suppression, derivation of approximate shear/extension rates, Fourier transform analysis of TEM images of 3D printed BCP nanostructures, stress relaxation experiments, images of extension-induced rheological instability, reduced end-on SM-SAXS visualizations of filament cross sections, and stress-strain behavior of 3D printed sheets at varied orientations­(PDF)
