# Supplementary material for: Microbeam SAXS Reveals Flow-Induced Nanostructural Heterogeneity and Tilted Domains in 3D-Printed Block Copolymers
Source: Macromolecules. 2026 Aug 13;59(16):8821–35. doi: 10.1021/acs.macromol.6c01113 (PMC13520875; doi:10.1021/acs.macromol.6c01113)
Supplement: Supplementary file 1 [file ma6c01113_si_001.pdf]

Supporting Information

Microbeam SAXS Reveals Flow-Induced Nanostructural Heterogeneity and Tilted Domains in 3D-Printed Block Copolymers

*Alice S. Fergerson, Emily C. Davidson**

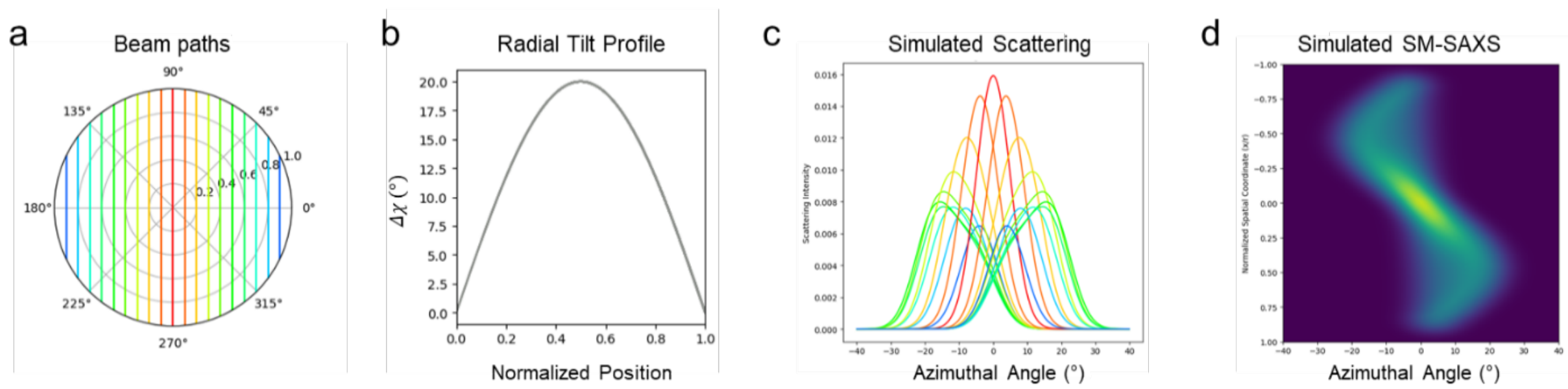

Figure S1. Simulation of SM-SAXS experiment for a whole filament by application of forward Abel transform. (a) Depiction of the set of vertical beam paths through a cylindrical filament. (b) Pre-defined radially-varied tilt profile $\Delta\chi(r)$. (c) Predicted scattering intensity from each scan; colors indicate relative position within the filament according to beam path colors in (a). (d) 2D visualization of the simulated SM-SAXS 'experimental' outcome for this defined $\Delta\chi(r)$ profile.

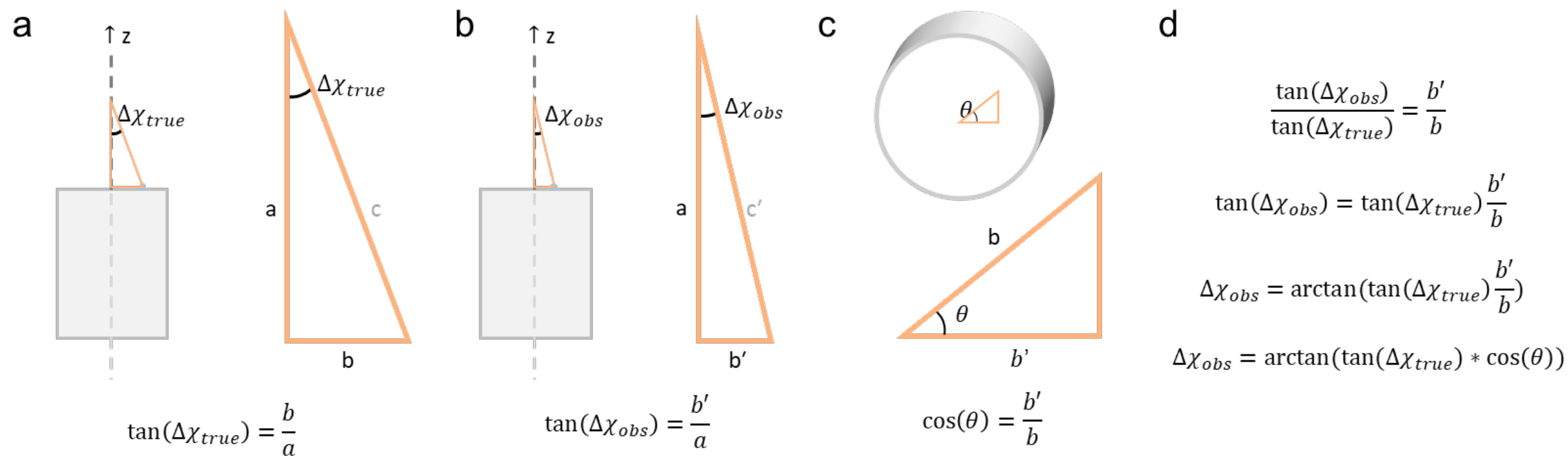

Figure S2. Schematic derivation of apparent tilt suppression for tilted scattering features. Here, the measured tilt angle for a domain at a radial distance *b* from the filament center and which is tilted at $\Delta\chi_{true}$ is being considered in all schematics. In all cases, the incoming X-ray beam is oriented within the plane of the filament cross-section, perpendicular to θ=0°. Figure S3 contains a schematic showing the definitions of the two angles referenced here. (a) Observation when θ=0° provides a relationship between $\Delta\chi_{true}$ and *b*. (b) Observation when θ≠0° provides a relationship between $\Delta\chi_{obs}$ and *b*'. (c) The polar angle θ provides a relationship between *b* and *b*'. (d) The relationships obtained from (a-c) provide the relevant relationship for tilt suppression between $\Delta\chi_{obs}$ and $\Delta\chi_{true}$. Figure S3 shows the actual tilt suppression effects resulting from this relationship.

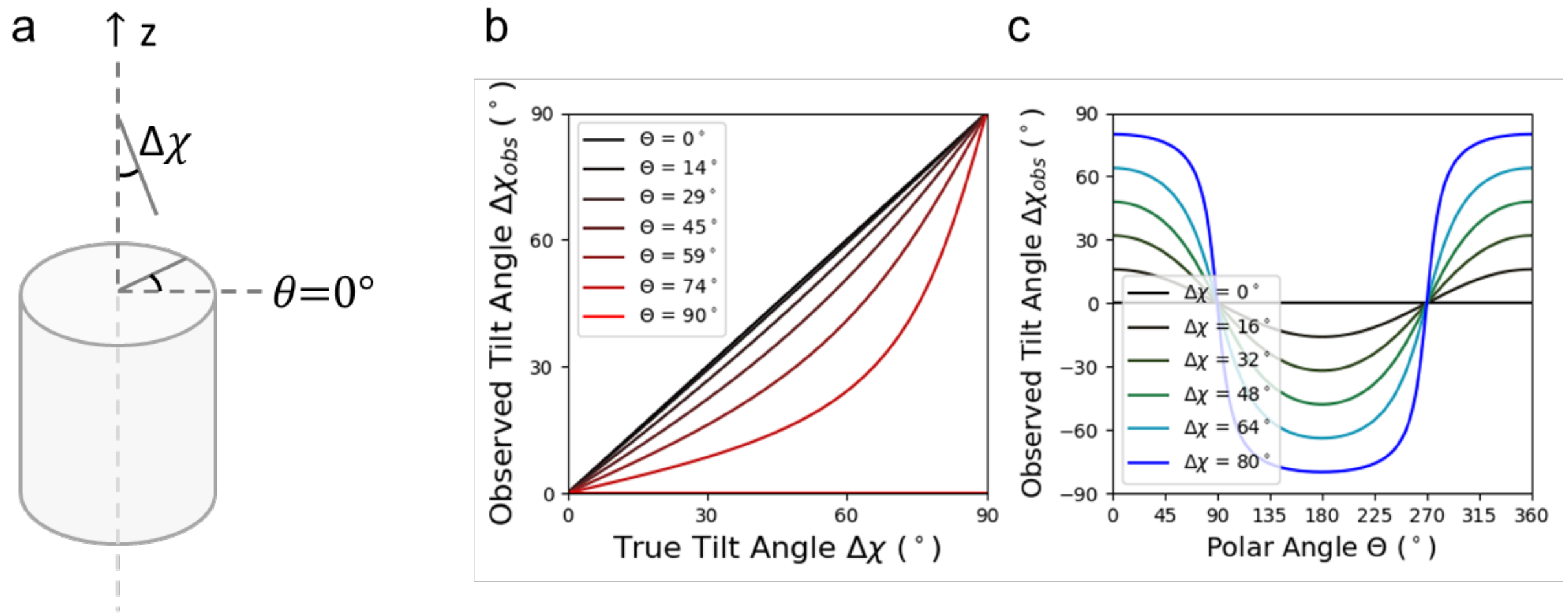

Figure S3. Suppression of the observed tilt of the scattering contribution of tilted domains in SM-SAXS. Suppression depends on the true tilt angle and the polar angle at which the measurement is taken. All plots here represent isolated analyses of how tilt is suppressed based on measurement angle for an individual tilted domain, and are not representative of complications arising from heterogeneous structure or beam path convolution. (a) Schematic defining key relevant angles, including the azimuthal angle $\Delta\chi$ representing observed/true tilt angles, and the polar angle θ. The incoming X-ray beam is oriented within the plane of the cross-section of the filament and is perpendicular to θ=0°. (b) Suppression of observed angle relative to the true tilt angle, shown for a series of polar angles. (c) Observed tilt angle as a function of polar angle, shown for a series of different true tilt angles.

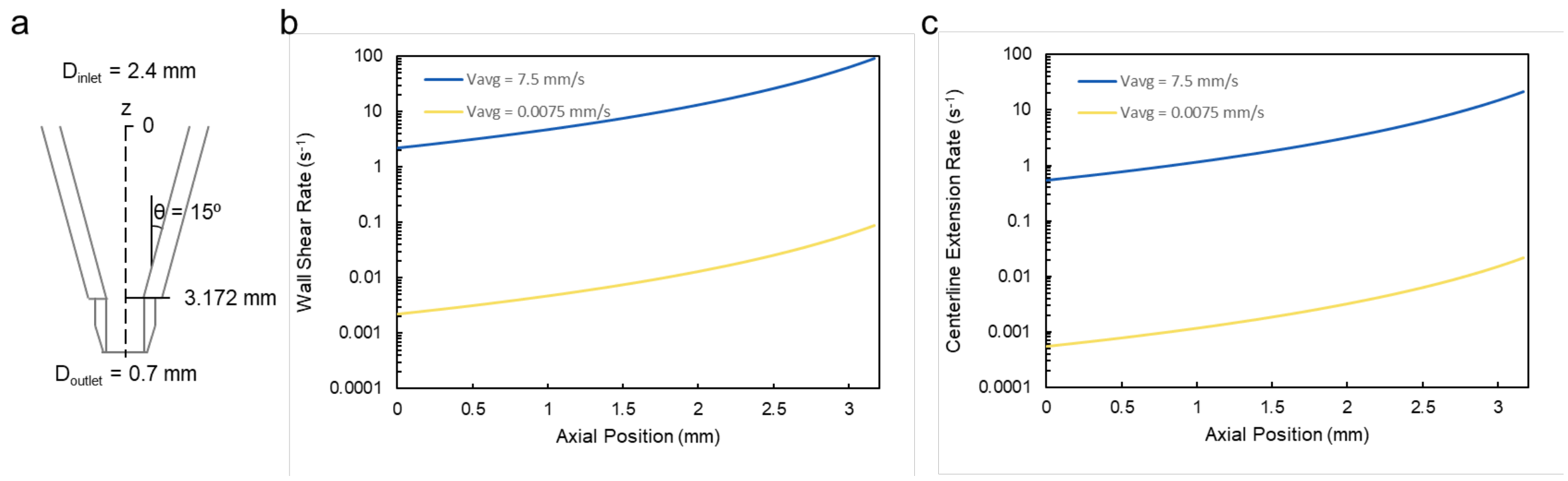

Figure S4. Approximation of shear and extension rates given a power law fluid model and assuming incompressibility, fully developed flow, and no-slip boundary conditions. (a) Conical nozzle geometry and relevant dimensions. Wall shear rate (b) and centerline extension rate (c) within converging region of a conical nozzle ($z \leq 3.172$ mm). Not plotted: wall shear rates remain constant over the cylindrical portion while extensional strain rates fall to zero given these assumptions.

Flow of a Power Law Fluid in a Conical Converging Nozzle:

Locally assume fully developed flow:

$$0 = -\frac{dP}{dz} + \frac{1}{r}\frac{d}{dr}(r\,\tau_{rz}) \qquad ; \qquad \eta = m\,\dot{\gamma}^{\,n-1}$$

The cross-sectional mean velocity is:

$$\bar{V} = \frac{Q}{\pi R(z)^2} = \frac{V_{\text{avg}}\,a^2}{R(z)^2},$$

Which leads to the Weissenberg-Rabinowitsch wall shear rate,

$$\dot{\gamma} = \left(\frac{3n+1}{n}\right)\frac{Q}{\pi R(z)^3} = \left(\frac{3n+1}{4n}\right)\frac{4\bar{V}}{R(z)} = \left(\frac{3n+1}{4n}\right)\frac{4\,V_{\text{avg}}\,a^2}{R(z)^3}.$$

Where a is the radius of the nozzle-exit and V_{avg} is the translation speed at $DR = 1$

So:

$$\dot{\varepsilon}(z) = \frac{d\bar{V}}{dz} = -\frac{2Q}{\pi R(z)^3}\frac{dR}{dz}$$

$\frac{dR}{dz} = -\tan\theta$ in the cone ($\theta =$ half-angle) and $\frac{dR}{dz} = 0$ in the straight cylindrical portion, so:

$$\dot{\varepsilon}(z) = \frac{2\,\bar{V}(z)}{R(z)}\,\tan\theta.$$

And,

$$V_{\text{max}} = \bar{V}\left(\frac{3n+1}{n+1}\right) \text{ so } \frac{dV_{\text{max}}}{dz} = \frac{d\bar{V}}{dz}\left(\frac{3n+1}{n+1}\right) \text{ and } \dot{\varepsilon}_{\text{center}} = \dot{\varepsilon}_{\text{avg}}\left(\frac{3n+1}{n+1}\right).$$

This gives expressions for $\dot{\gamma}_{\text{wall}}(z)$ and $\dot{\varepsilon}_{\text{center}}(z)$:

$$\dot{\gamma}_{\text{wall}}(z) = \left(\frac{3n+1}{n}\right)\frac{V_{\text{avg}}\,a^2}{R(z)^3},$$

$$\dot{\varepsilon}_{\text{center}}(z) = \left(\frac{3n+1}{n+1}\right)\frac{2\,V_{\text{avg}}\,a^2}{R(z)^3}\,\tan\theta,$$

With, for our nozzle geometry (where z = 0 at the nozzle top entrance)

$$R(z) = 1.2 - \tan(15^\circ)\,z \qquad (z = 0 \text{ to } 3.17 \text{ mm}),$$

where $a = 0.35$ mm is the exit radius and the entrance radius is $R(0) = 1.2$ mm.

Note that over the cylindrical portion, the shear rate is constant, while the extension rate is zero.

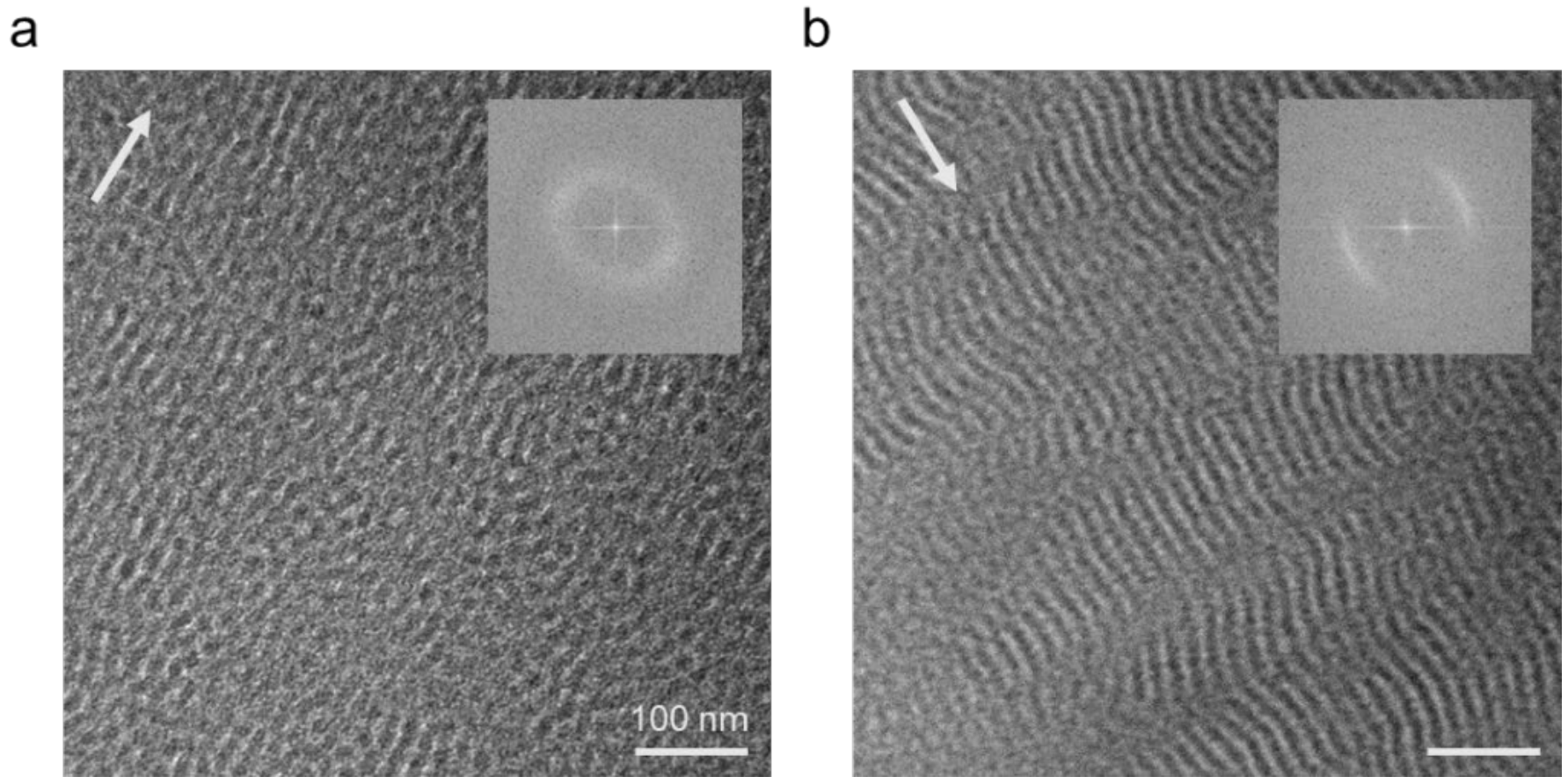

Figure S5. TEM images of 3D printed BCPs with inset Fourier transforms. Micrographs show(a) Short, broken-cylinder nanostructure observed in a sample printed using v_{avg} = 7.5 mm/s, D_R = 1, prior to thermal annealing. (b) Cylindrical nanostructure observed in a sample printed using v_{avg} = 7.5 mm/s, D_R = 1, after thermal annealing. Inset Fourier transforms highlight that the apparent degree of orientation as would be observed via SAXS is strongly impacted by the extent of short-range cylinder connectivity. Original TEM images adapted with permission.[1] Copyright Wiley, 2024.

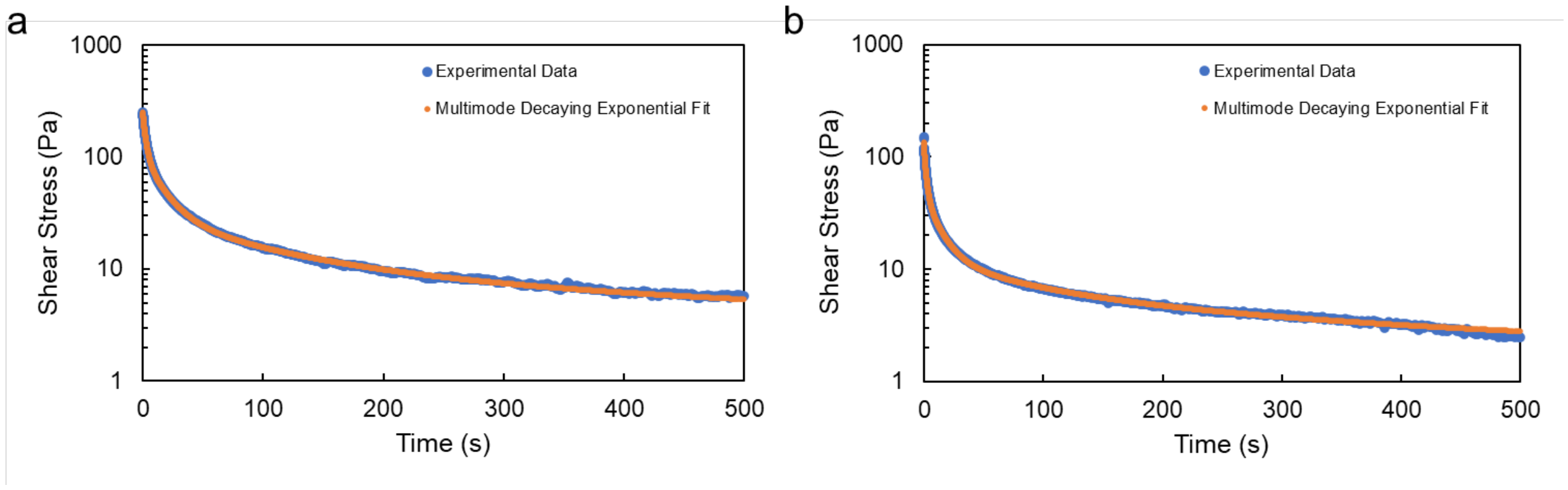

Figure S6. Stress relaxation and multimode decaying exponential fits for SEBS melts after application of steady shear to a total shear strain of 3200% at fixed shear rates of (a) 0.1 s^{-1} and (b) 1 s^{-1}. Average extracted relaxation times are (a) 16 s, and (b) 15 s.

Shear rate (s^{-1})	i	1	2	3	4	5	b	avg
0.1	a_i (Pa)	53.76	131.37	71.36	17.64	9.45	3.55	-
	λ_i (s)	0.168	2.71	16.3	80.9	301	-	16.43
1	a_i (Pa)	47.76	65.90	32.41	6.12	5.02	1.88	-
	λ_i (s)	0.166	2.07	12.6	67.1	295	-	15.5

Table S1. Relaxation times and weighting coefficients from multimode decaying exponential fit of the form $\tau(t) = \sum_i a_i e^{-t/\lambda_i} + b$, with weighted average relaxation time calculated according to: $\lambda_{avg} = \frac{\sum a_i \lambda_i}{\sum a_i}$, following literature precedent.[2]

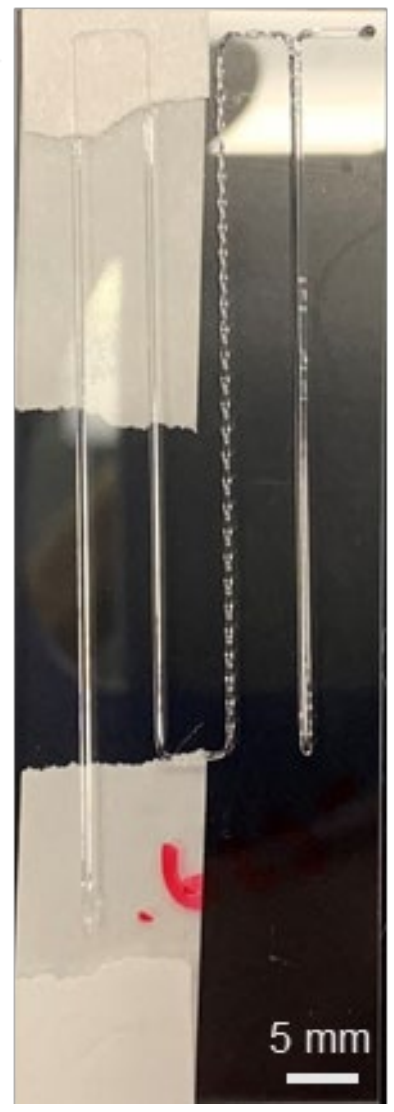

Figure S7. Extension-induced rheological instability. Rheological instability in 3D printed SEBS which can emerge at high draw ratio conditions. Similar rheological instabilities have been leveraged as a tunable flow parameter in 3D printing[3].

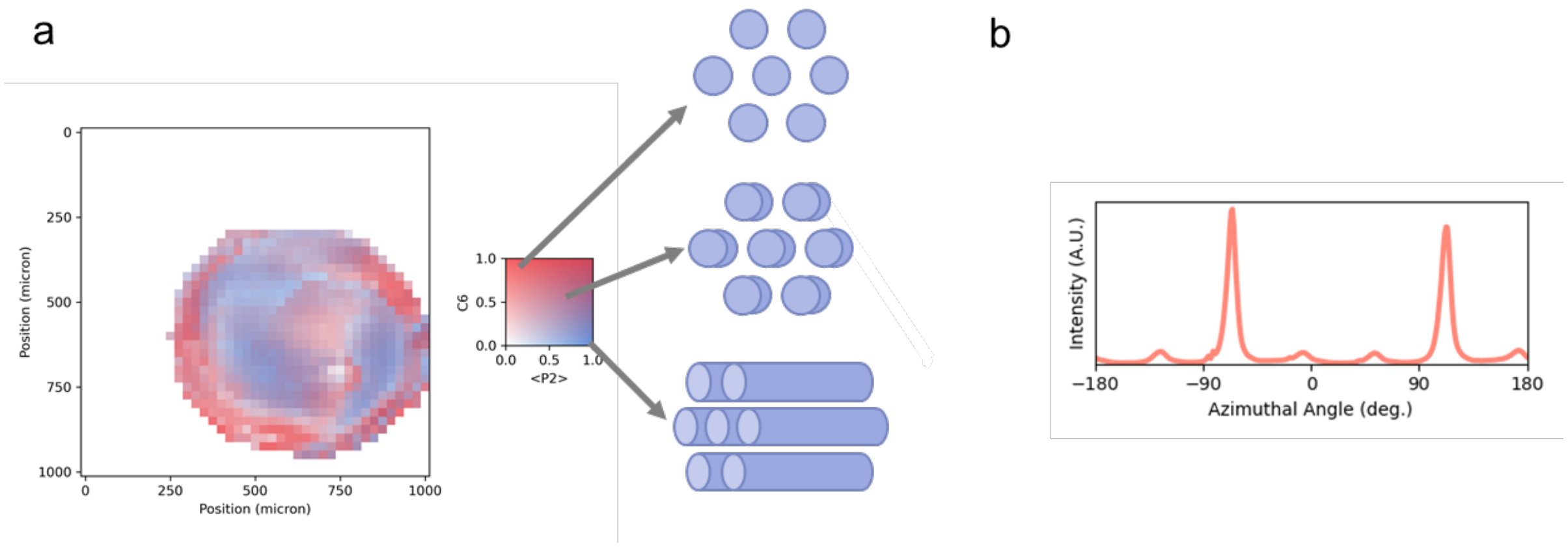

Figure S8. Convolution of 2- and 6-fold symmetry in 'end-on' filament mapping scans. (a) Combined visualization of 2-fold and 6-fold order parameters extracted from an area-mapping scan of a filament cross-section. Schematics on the right represent the orientations represented by individual or 'mixed' order parameter signals. (b) 1-D reduced I(χ) scattering profile for one such 'mixed' identified order parameter. Suppression of four of six scattering peaks leads to difficulty in distinguishing a tilted region from the combined signal from two differently oriented domains.

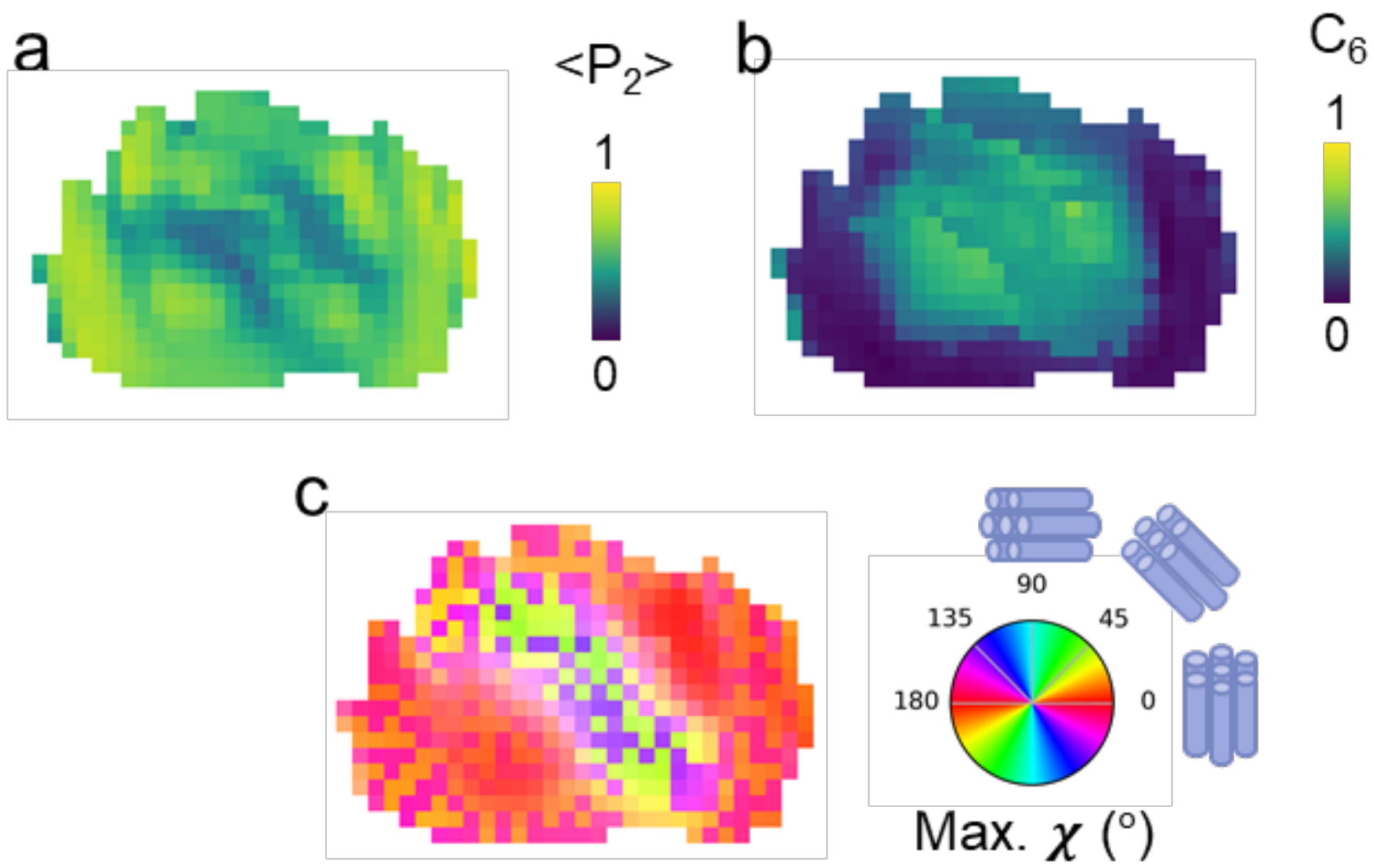

Figure S9. End-on SM-SAXS characterization of a high draw ratio filament cross-section. (a) Extent of calculated two-fold symmetry is high as a result of slight macroscopic sample tilting, resulting in the phenomenon shown in Figure S5b. (b) Extent of 6-fold symmetry from domains without significant off-axis tilting is high and persists through a large 'core' region of the filament. (c) Spatial map of the same filament showing the angular position of the azimuthal maximum in scattering intensity. For any apparent 2-fold orientation as identified in (a), the schematics on the right indicate the appropriate direction of nanostructure tilting or orientation. The overall orange and red coloration throughout the region identified as exhibiting a high degree of 2-fold symmetry in (a) is evidence of a slight macroscopic sample tilt to yield this suppression in scattering during the measurement, rather than a widespread presence of true cylinder orientation 'vertically' within the plane of the cross-section. The 'jagged' edges of these visualizations stem from challenges associated with the use of automated intensity cutoffs to distinguish scans which pass through the cross section from scans which exclusively contain streaks caused by filament surface reflections.

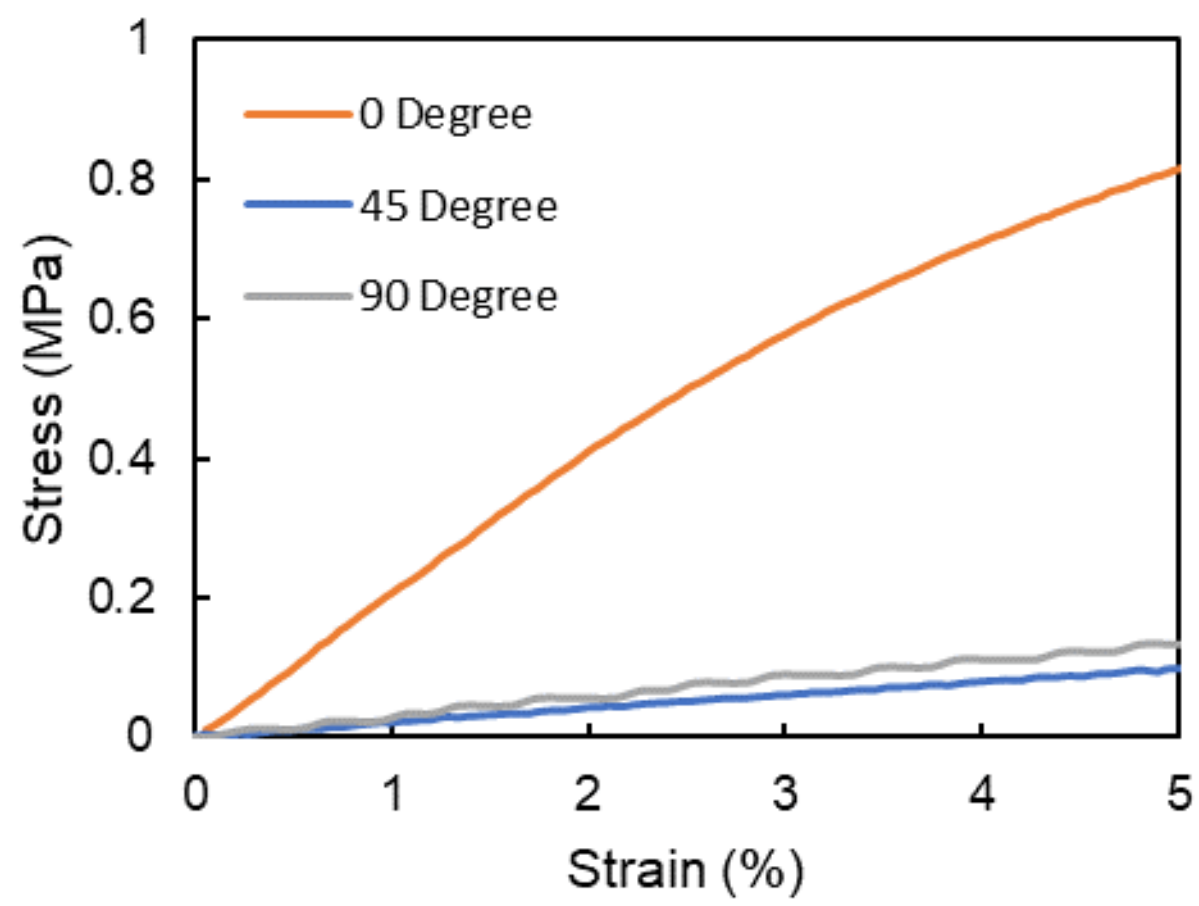

Figure S10. Stress-strain curves for sheets strained at three orientations relative to the print direction. Extracted small strain moduli are used to fit stress tensor coefficients in Equation 5.

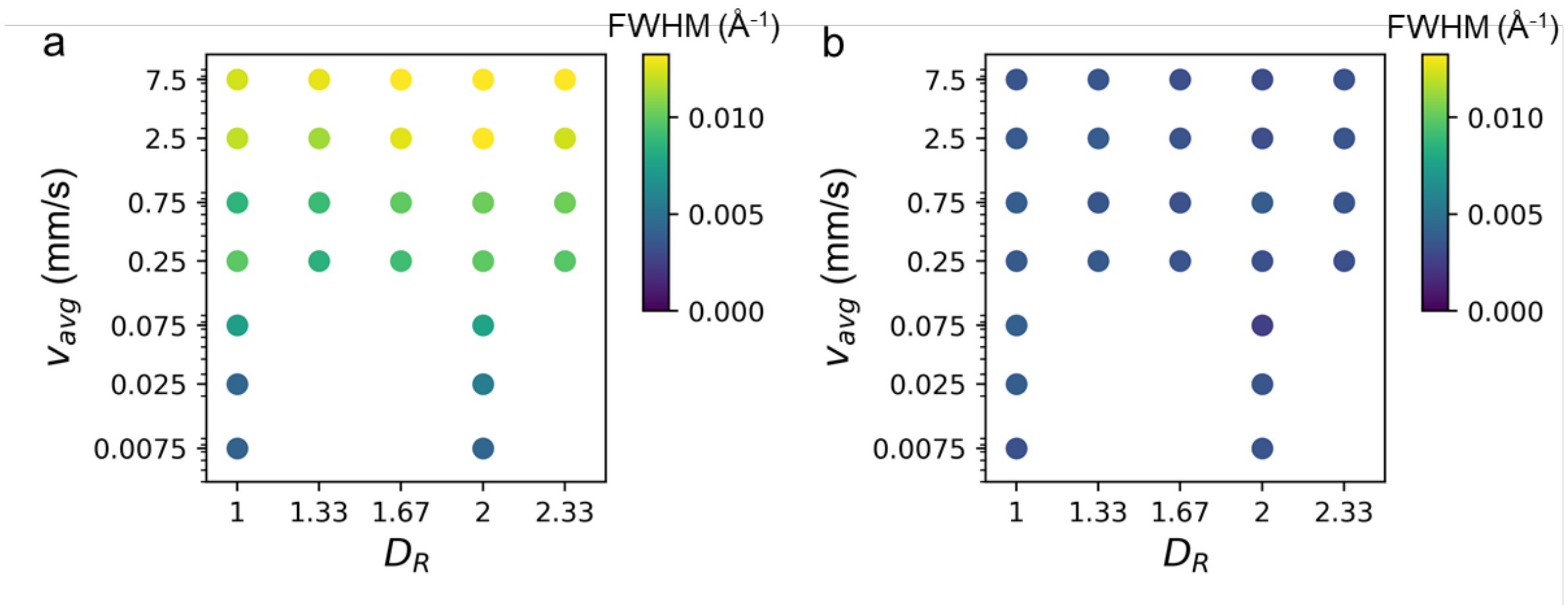

Figure S11. FWHM of the primary scattering peak (q = 0.025 – 0.04 Å^{-1}) (a) upon 3D printing and (b) after thermal annealing. Significant peak broadening occurs prior to annealing at high v_{avg} and high D_R conditions. The magnitude of the nanostructural relaxation associated with thermal annealing appears to correlate with the recovery of long-range domain continuity in this 3D printed block copolymer.

References

(1) Fergerson, A. S.; Gorse, B. H.; Maguire, S. M.; Ostermann, E. C.; Davidson, E. C. Reprocessable and Mechanically Tailored Soft Architectures Through 3D Printing of Elastomeric Block Copolymers. *Adv Func Mater* **2024**, *34* (48), 2411812. https://doi.org/10.1002/adfm.202411812.

(2) McCready, E. M.; Burghardt, W. R. In Situ SAXS Studies of Structural Relaxation of an Ordered Block Copolymer Melt Following Cessation of Uniaxial Extensional Flow. *Macromolecules* **2015**, *48* (1), 264–271. https://doi.org/10.1021/ma501633f.

(3) Dreier, L.; Marzin, T.; David, R.; Pienaar, S.; Eujayl, A.; Vogeley, R.; Xi, Y.; Brun, P.-T. Liquid Lace: Regular Pattern Formation by under-Extrusion. *Phys. Rev. Fluids* **2024**, *9* (11), 110512. https://doi.org/10.1103/PhysRevFluids.9.110512.
